# Validity and Reliability of Inertial Measurement Units on Lower Extremity Kinematics During Running: A Systematic Review and Meta-Analysis

**DOI:** 10.1186/s40798-022-00477-0

**Published:** 2022-06-27

**Authors:** Ziwei Zeng, Yue Liu, Xiaoyue Hu, Meihua Tang, Lin Wang

**Affiliations:** School of Kinesiology, Shanghai University of Sport, Shanghai, China

**Keywords:** Inertial measurement unit, Kinematics, Running, Validity, Reliability

## Abstract

**Background:**

Inertial measurement units (IMUs) are useful in monitoring running and alerting running-related injuries in various sports settings. However, the quantitative summaries of the validity and reliability of the measurements from IMUs during running are still lacking. The purpose of this review was to investigate the concurrent validity and test–retest reliability of IMUs for measuring gait spatiotemporal outcomes and lower extremity kinematics of health adults during running.

**Methods:**

PubMed, CINAHL, Embase, Scopus and Web of Science electronic databases were searched from inception until September 2021. The inclusion criteria were as follows: (1) evaluated the validity or reliability of measurements from IMUs, (2) measured specific kinematic outcomes, (3) compared measurements using IMUs with those obtained using reference systems, (4) collected data during running, (5) assessed human beings and (6) were published in English. Eligible articles were reviewed using a modified quality assessment. A meta-analysis was performed to assess the pooled correlation coefficients of validity and reliability.

**Results:**

Twenty-five articles were included in the systematic review, and data from 12 were pooled for meta-analysis. The methodological quality of studies ranged from low to moderate. Concurrent validity is excellent for stride length (intraclass correlation coefficient (ICC) (95% confidence interval (CI)) = 0.937 (0.859, 0.972), *p* < 0.001), step frequency (ICC (95% CI) = 0.926 (0.896, 0.948), *r* (95% CI) = 0.989 (0.957, 0.997), *p * < 0.001) and ankle angle in the sagittal plane (*r* (95% CI) = 0.939 (0.544, 0.993), *p* = 0.002), moderate to excellent for stance time (ICC (95% CI) = 0.664 (0.354, 0.845), *r* (95% CI) = 0.811 (0.701, 0.881), *p* < 0.001) and good for running speed (ICC (95% CI) = 0.848 (0.523, 0.958), *p* = 0.0003). The summary Fisher's Z value of flight time was not statistically significant (*p* = 0.13). Similarly, the stance time showed excellent test–retest reliability (ICC (95% CI) = 0.954 (0.903, 0.978), *p* < 0.001) and step frequency showed good test–retest reliability (ICC (95% CI) = 0.896 (0.837, 0.933), *p* < 0.001).

**Conclusions:**

Findings in the current review support IMUs measurement of running gait spatiotemporal parameters, but IMUs measurement of running kinematics on lower extremity joints needs to be reported with caution in healthy adults.

*Trial Registration*: PROSPERO Registration Number: CRD42021279395.

**Supplementary Information:**

The online version contains supplementary material available at 10.1186/s40798-022-00477-0.

## Key Points

- IMUs are reliable tools for measuring gait spatiotemporal parameters during running in healthy adults but should be reported with caution for lower extremity joint kinematics.
- Future studies need to include more subjects and use more rigorous protocols to provide evidence that supports the use of IMUs in the prevention of running-related injuries.
- Guidelines for applying IMUs for running kinematic measurement need to be established.

## Introduction

Running is one of the popular physical activities around the world and has positive effects on both physical and mental health [1, 2]. Unfortunately, overuse due to the increased frequency and volume of running is the main mechanism for the occurrence of running-related injuries (RRIs), particularly in the lower limbs [3–7]. Therefore, a thorough understanding of biomechanical changes in the lower limbs during running is of great importance to the prevention of RRIs.

As a portable alternative to optical motion capture systems, inertial sensors are becoming increasingly popular in many fields, including sports science, owing to their low cost, portability, lightness and unlimited research environment [8–11]. Inertial sensors usually include accelerometers, gyroscopes or magnetometers, also known as inertial measurement units (IMUs), which measure gravitational acceleration, angular velocity and heading in the Earth’s magnetic field, respectively [12, 13].

Along with the increasing popularity of IMUs, the number of studies examining their validity and reliability for a variety of populations (e.g., healthy people, multiple sclerosis and stroke patients) during different movements (e.g., walking, running and jumping) has increased [14–16]. Recent systematic reviews have examined the validity and reliability of measurements from IMUs of walking in healthy adults [17] and the impact of IMUs’ position on the validity and reliability of stride variables during running [18]. However, three-dimensions kinematics data for the validity and reliability of measurements from IMUs during running have not been synthesized and quantified. Meanwhile, limiting the study population to healthy adults may render the findings more homogeneous. Therefore, the aim of the current systematic review and meta-analysis was to determine the concurrent validity and test–retest reliability of IMUs for measuring gait spatiotemporal and lower-extremity kinematics outcomes during running in healthy adults.

## Methods

The protocol was registered on the International Prospective Register of Systematic Reviews (PROSPERO) (Registration number: CRD42021279395) and followed the Preferred Reporting Items for Systemic Reviews and Meta-Analysis (PRISMA) guidelines [19].

### Search Strategy

PubMed, CINAHL, Embase, Scopus and Web of Science electronic databases were searched from inception until 27 September 2021. The search terms and strategies included: (wearable sensor* OR inertial sensor* OR inertial motion capture OR "Wearable Electronic Devices"[Mesh] OR inertial measurement unit* OR IMU OR "Micro-Electrical–Mechanical Systems"[Mesh] OR MEMS OR acceleromet* OR gyroscop* OR magnetomet* OR smart phone OR "Smartphone"[Mesh]) AND (running speed OR cadence OR (step frequency) OR (stride frequency) OR (step time) OR (stride time) OR (cycle time) OR (contact time) OR (swing time) OR (flight time) OR (step length) OR (stride length) OR spatiotemporal OR "Spatio-Temporal Analysis"[Mesh] OR kinematic* OR biomechanic* OR (joint angle) OR hip OR knee OR ankle OR range of motion OR "Range of Motion, Articular"[Mesh]) AND (running OR jogging OR sprinting) AND (validity OR reliability OR feasibility OR repeatability OR consistency OR "Reproducibility of Results"[Mesh] OR "Data Accuracy"[Mesh]). Minor adjustments were made for different databases. Full search strategies for each database can be found in Additional File 1.

### Inclusion and Exclusion Criteria

Articles that met the following criteria were included in this systematic review: (a) evaluated the validity or reliability of IMUs, (b) measured specific gait spatiotemporal and lower extremity kinematics parameters, (c) compared the measurements captured by IMUs with those obtained using reference systems, (d) collected data during running, jogging or sprinting, (e) assessed human beings and (f) were published in English. Any studies that only measured activity/movement identification or energy expenditure were excluded from this review. Additional details on the inclusion and exclusion criteria and definitions for the spatiotemporal parameters can be found in Additional File 2.

### Study Selection

After duplicate articles were removed, two independent reviewers (Zeng and Liu) screened the titles and abstracts according to the eligibility criteria. The full-text screening of the potentially eligible articles was examined by one author (Zeng) and rechecked by a second author (Hu). All reference lists and bibliographies of the retrieved studies were reviewed in case relevant studies were missed by the electronic search. Disagreements were discussed and resolved by a third reviewer (Wang).

### Assessment of Risk of Bias

Assessment of risk of bias was assessed using a modified version of the Critical Appraisal of Study Design for Psychometric Articles [20], which was adjusted by Kobsar et al. [17] to specifically evaluate the psychometric properties of studies about inertial sensors. This checklist contains 12 items, which assess the methodological quality of five domains, namely, study question, study design, measurements, analyses and recommendations [17]. Each item comprises three descriptors. The maximum score is 24 and the final total score and percentage will be presented. Initially, two assessors (Tang and Liu) reviewed two articles at the same time, and then a consensus on the scoring and interpretation of each item was performed before the remaining articles were evaluated separately. The process described above in case of disagreement was used. Assessors were blinded to any identifiable information related to the studies to avoid bias in quality assessment. Furthermore, agreement between the two assessors was calculated using the Cohen’s kappa coefficient with a 95% confidence interval (95% CI) [21]. Cohen’s kappa coefficient of < 0.40, 0.40–0.75 or > 0.75 were regard as poor, fair to good or excellent, respectively [22].

To grade the quality of the study, a previously described classification scheme was applied (Table 1) [17]. Quality assessment scoring was then used in determining the strength of recommendations [23].

**Table 1:** Study Methodological quality grading scheme [17]

Level	Score
High quality (HQ)	85–100%
Moderate quality (MQ)	70–85%
Low quality (LQ)	50–70%
Very low quality (VLQ)	< 50%

### Data Extraction

Data extraction was completed by two authors (Zeng and Tang) using a pre-defined form. The data consisted of (1) study identification information; (2) participant characteristics: sample size, sex, age, height, weight and recruited population; (3) IMUs’ specifications: name, manufacturer, composition, used number, placement and sample frequency; (4) reference systems used; (5) study design: running speed/running distance and research field; (6) specific parameters; and (7) reported statistical outcomes.

For validity, statistical outcomes extracted were Pearson correlation coefficient (*r*), coefficient of determination (r^2^), coefficient of multiple correlation (CMC), concordance correlation coefficient (CCC) and intraclass correlation coefficient (ICC) with 95% CI, root mean square error (RMSE; measurement error between the IMUs and reference systems), bias (mean difference between the IMUs and reference systems) and limits of agreement (LoA; 1.96*standard deviation of the difference between the IMUs and reference systems). For reliability, the statistical outcomes extracted were ICC (95% CI), RMSE, coefficient of variation (CV; the ratio of the standard deviation to the mean), and standard error of the mean. It should be mentioned that ICCs were not included in this review if they were only shown graphically without specific values and all differences were unified as the IMUs’ measurement minus the reference systems’ measurement if mentioned in the articles. While these statistical outcomes were extracted for the qualitative assessments, data pooling was a priori set to assess only the ICCs, *r* and sample size for validity and ICCs and sample size for reliability.

### Statistical Analysis

In data pooling, validity and reliability were first dichotomised. Then, a further division was made among specific parameters. Given that reported ICC and *r* values need to be classified, a single study may contribute to multiple independent data pooling based on validity, reported statistical outcomes and parameter measured. The agreement metrics of ICCs were interpreted as poor (< 0.500), moderate (0.500–0.749), good (0.750–0.899) or excellent (≥ 0.900) [24], and *r* was interpreted as no correlation (< 0.250), fair relationship (0.250–0.500), moderate to good relationship (0.500–0.750) or good to excellent relationship (≥ 0.750) [25].

Review Manager (RevMan 5.3) was used in the meta-analysis. Heterogeneity was examined using Tau^2^, Chi^2^ and I^2^ statistics where Tau^2^ = 0 suggests no heterogeneity; I^2^ values of < 25%, 26%–50% and > 75% suggest low, moderate and high heterogeneity, respectively, and a significant Chi^2^ indicates heterogeneity [26]. The level of significance was *P* < 0.05. Due to the heterogeneity of the experimental conditions and population, a random effects model was used with 95% CI [27]. When the number of studies is sufficient (*n* ≥ 3), subgroup analyses were conducted to explore the potential sources of heterogeneity. Subgroup were defined a priori and included running speed, IMUs’ position and running surface. The running speed was set to two levels: low (speed ≤ 15 km/h) and fast (speed > 15 km/h), and the running surface was divided into treadmill and ground. Sensitivity analyses were performed by deleting one study at a time to evaluate the stability of the results [28].

Weighting of individual point estimates was based on sample size. Given the non-normality of ICCs and *r*, point estimates were variance-stabilised using Fisher’s z-transformation as follows [29]:

1 $${\mathrm{Fisher}}^{\mathrm{^{\prime}}}\mathrm{s }\,{\mathrm{Z}}_{\mathrm{ICC}}=0.5\times \mathrm{ln}\frac{1+\mathrm{ICC}}{1-\mathrm{ICC}}$$

2 $${\mathrm{Fisher}}^{\mathrm{^{\prime}}}\mathrm{s }\,{\mathrm{Z}}_{r}=0.5\times \mathrm{ln}\frac{1+r}{1-r}$$

3 $${v}_{z}=\frac{1}{n-3}$$

4 $${\mathrm{SE}}_{\mathrm{ICC}}=\frac{1}{\sqrt{n-3/2}}$$

5 $${\mathrm{SE}}_{r}=\sqrt{{v}_{z}}$$

6 $$\mathrm{Summary} \,\text{ICC}/r=\frac{{\mathrm{e}}^{2\mathrm{Z}}-1}{{\mathrm{e}}^{2\mathrm{Z}}+1}$$

where n represents sample sizes, SE depicts standard error and Z is Summary Fisher's Z value [30]. Data were then transformed back to ICCs or *r* for reporting. The results of the meta-analysis were interpreted using the same agreement metrics outlined above.

Statistical results that were not included in the quantitative analysis were included in the qualitative analysis to support the interpretation. An adapted rating system from the Cochrane collaboration back review group [23] was used in determining the level of evidence for each parameter (Table 2) [17, 31].

**Table 2:** Definitions of levels of evidence [17]

Level of evidence	Criteria
Strong evidence	Consistent results in HQ studies (*n* ≥ 2)
Moderate evidence	Consistent results among multiple MQ studies (*n* ≥ 2)
Limited evidence	Consistent results among multiple LQ studies (*n* ≥ 2)
Conflicting evidence	Inconsistent results among multiple studies
Very limited evidence	Only one LQ or MQ study or multiple VLQ studies

*HQ* high-quality, *MQ* moderate-quality, *LQ* low-quality, *VLQ* very low quality

## Results

### Characteristics of the Included Studies

**Fig. 1:**
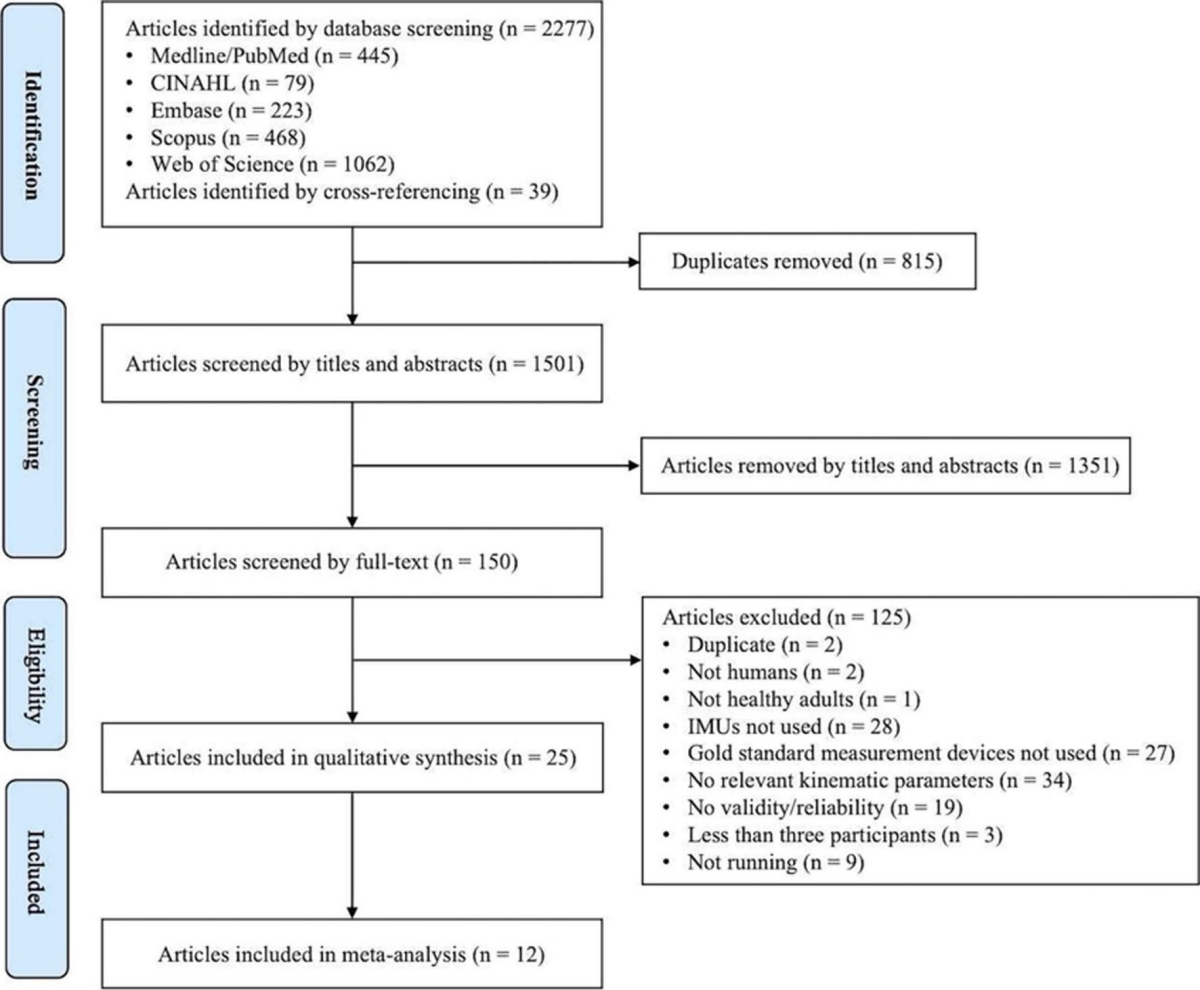
Flowchart of the systematic review selection process.

A total of 2316 articles were identified through database screening and cross-referencing. After the removal of duplicates, screening of titles and abstracts, and full-text screening, 25 studies met the eligibility criteria and were included in this systematic review [12, 32–55]. An outline of the screening process using the PRISMA flow diagram is presented in Fig. 1.

A summary of the characteristics of the included 25 studies is presented in Table 3. A total of 541 healthy adults (mean [sd] sample size: 22 [12] participants; range: 7–51 participants; 151 females and 354 males; sex was not described in Fox et al. [41] and Li et al. [48]) were included in this review. In terms of the population, it has been divided it into four categories, namely competitive runners (*n* = 111) [32, 33, 36, 37, 51, 52], experienced runners (*n* = 28) [43, 53], amateur runners (*n* = 200) [33, 41, 42, 45, 47, 54, 55] and non-runners (*n* = 202) [12, 34, 35, 38–40, 44, 46, 48–50]. The most common IMU systems used were the Xsens system (*n* = 3) [12, 34, 53] and RunScribe™ system (*n* = 3) [38, 42, 45]. Using two (*n* = 9) [32, 35, 40–42, 44, 45, 51, 54] or one (*n* = 8) [33, 34, 36, 37, 43, 46, 47, 52] IMU was the most preferable, and some studies used five (*n* = 1) [49], seven (*n* = 3) [39, 48, 50], eight (*n* = 1) [50] or seventeen (*n* = 2) [12, 53] IMUs. In addition, studies installed IMUs in diverse sites, including dorsum of the foot [12, 32, 34, 38–40, 42, 44, 45, 49, 50, 53, 55], ankle [38, 48, 51, 55], heel [38, 47, 55], shank [12, 35, 38, 39, 44, 49, 50, 53], knee [48], thigh [12, 35, 39, 50, 53], hip [46, 48], waist [36, 37, 43, 53], sacrum [12, 49, 50], chest [38, 41], sternum [12, 52, 53], back [33, 39, 41], upper arm [12, 53], lower arm [12, 53], hand [12, 53], shoulder [12, 53], head [12, 53] and shoes midsole [54, 55]. The most common sampling frequencies used in assessing running were 200 Hz (*n* = 6) [33, 41, 45, 49, 54, 55] and 500 Hz [36, 37, 40, 42, 43] (*n* = 5; range: 50–1000 Hz). For the sports settings, the present study included running on an indoor track or walkway, running on a treadmill and running outside, and running speed ranged from 7.2 km/h to 21 km/h.

**Table 3:** Study characteristics

Author(s), Year [Reference No.]	Participant (size, age, height, weight, population)	IMUs					Reference system	Running speed/running distance	Research field	Parameters
		Name (manufacturer)	Composition	Number	Placement	Sample frequency				
Ammann et al., 2016 [32]	12 subjects (5 F, 7 M; age: 25.3 ± 3.2 years; height: 174.4 ± 7.9 cm; weight: 64.8 ± 10.2 kg) High-level running athletes	PARTwear (PW, HuCE-microLab, University of Applied Sciences, Biel, Switzerland)	3D accelerometer (± 16 g); 3D gyroscope; 3D magnetometer	2	Lace of the shoe	1000 Hz	OMC (Camera Marathon Ultra CL600, Videal AG, Niederonz, Switzerland)	Maximal sprinting speed (8.0 ± 0.5 m/s); intense training speed (6.2 ± 0.7 m/s); normal training speed (4.3 ± 0.7 m/s); all speeds (6.2 ± 1.6 m/s) (40 m)	Indoor track	Stance time
Bergamini et al., 2012 [33]	Group A: 6 amateur athletes (2F, 4 M; height: 172 ± 12 cm; weight: 63.50 ± 10.84 kg) Group E: 5 elite athletes (2F, 3 M; height: 177 ± 76 cm; weight: 65.00 ± 8.25 kg) Total: 11 participants (4F, 7 M; height: 174 ± 10 cm; weight: 64.18 ± 9.78 kg)	IMU (FreeSense, Sensorize, Italy)	3D accelerometer (± 6 g); 3D gyroscope (± 500°/s)	1	Lower back trunk (L1 level)	200 HZ	OMC (Casio Exilim EX-F1, Japan); 9 force platforms (Z20740AA, Kistler, Switzerland)	Three sprint runs of 60 m	Indoor track; outdoor training track	Stance time, stride time
Brahms et al., 2018 [34]	11 healthy young adults (4F, 7 M; age: 22.3 ± 1.5 years; height: 175.2 ± 23.1 cm; weight: 76.04 ± 3.19 kg)	Xsens (MTw)	–	1	Right foot	100 Hz	OMC (Vicon)	A range of typical distance running speeds/10 m trial runs (3.55 ± 0.34 m/s, range: 2.71–4.36 m/s)	Indoor	Stride length
Cooper et al., 2009 [35]	7 subjects (2F, 5 M; age: 30 ± 6 years; height: 170 ± 20 cm; weight: 70 ± 11 kg)	IMU (ETB Ltd, Codicote, UK)	3D accelerometer (± 5 g); 3D gyroscopes (± 1200°/s)	2	Thigh and shank	100 Hz	OMC (Qualysis)	5 mile/hour	Treadmill	Knee joint flexion/extension angles
Day et al., 2021 [36]	30 subjects (21F; weight: 54.0 ± 5.3 kg; 9 M; weight: 63.6 ± 6.7 kg) National Collegiate Athletic Association Division 1 cross country runners	IMU (IMeasureU, Auckland, New Zealand)	–	1	Over waistband	500 Hz	Instrumented treadmill (Treadmetrix, Park City, UT/Bertec, Columbus, OH)	M: 3.8, 4.1 and 5.4 m/s; F: 3.8 and 4.9 m/s	Treadmill	Stance time
Deflandre et al., 2018 [37]	Reliability: 10 young male athletes (age: 14 ± 0.5 years; height: 168 ± 7 cm; weight: 56 ± 9 kg) Validity: 20 male athletes (age: 32 ± 14 years; height: 181 ± 16 cm; weight: 71 ± 7 kg)	Myotest (Myotest SA, Sion, Switzerland)	3D accelerometer	1	The iliac crests mark, in the alignment with the umbilicus	Hz	The Optogait (Microgate, Bolzano, Italy); 3D optoelectronic CX1 units (Codamotion, Charnwood Dynamics Ltd, UK)	8 km/h, 12 km/h and 16 km/h (80 m); 8 km/h and 16 km/h	Outside: artificial turf field; indoor: treadmill	Stance time, step length, stride length, step frequency
De Fontenay et al., 2020 [38]	32 healthy participants (13F, 19 M; age: 27.0 ± 5.5 years; height: 174.4 ± 8.5 cm; weight: 69.1 ± 11.4 kg) 32 participants were analyzed for RunScribeTM, 31 participants were analyzed for MilestonePod and TgForce, 30 for Zoi, and 25 for Moov NowTM	Moov Now™ (Moov, San Mateo, California, USA); MilestonePod (Milestone Sports, Long beach, California, USA); RunScribe™ (Montara, California, USA); Zoi (Runteq, Tampere, Finland); TgForce (Kelsec Systems Inc.,Montréal, Canada)	–	–	Moov Now™: outside of the ankle and the loop end of the band forward; MilestonePod: shoelaces; RunScribe™: heel mount; Zoi: chest strap and shoelaces; TgForce: medial end of tibia	–	OMC (Vicon); Instrumented treadmill with force plates (Bertec, Columbus, OH, USA)	-	Treadmill	Step frequency
Dorschky et al., 2019 [39]	10 healthy male subjects (age: 27.1 ± 2.6 years; height: 182 ± 5 cm; weight: 76.9 ± 8.6 kg)	Custom-built IMUs (Portabiles GmbH, Erlangen, DE)	3D accelerometers (± 16 g); Gyroscopes (± 2000°/s)	7	Lower back, right and left lateral thigh, lateral shank, and upper midfoot	1000 Hz	OMC (Vicon MX, Oxford, UK); Force plate (Kistler Instruments Corp, Winterhur, CH)	3.0–4.9 m/s	Indoor	Hip, knee, and ankle angles in the sagittal plane
Falbriard et al., 2018 [40]	41 healthy adults (13F, 28 M; age: 29 ± 6 years; height 174 ± 8 cm; weight 70 ± 10 kg)	IMU (Physilog 4, Gait Up, Switzerland)	3D accelerometer (± 16 g); 3D gyroscope (± 2000 ◦/s)	2	Dorsum of each foot	500 Hz	Instrumented treadmill (T-170-FMT, Arsalis, Belgium)	Starting at 8 km/h and increasing by 2 km/h up to maximum speed	Treadmill	Stance time, flight time, swing time, step time
Fox et al., 2019 [41]	26 recreationally active participants (age: 32.2 ± 11.0 years; height: 173.3 ± 9.9 cm; weight: 74.2 ± 16.2 kg)	Polar Team Pro Sensor (Polar Electro, Kempele, Finland)	GPS; accelerometer; gyroscope; digital compass	2	Upper-torso between the scapulae and the centre of the chest at the level of the xiphoid process	200 Hz	Electronic timing light (Fusion Sport, Coopers Plains, QLD, Australia)	Medium (moderate, jogging) speed; high (maximal, sprint) speed	Indoor (sprung hardwood floor)	Running speed
García-Pinillos et al., 2019 [42]	49 amateur endurance runners (5F, 44 M; age: 26 ± 8 years; height: 174 ± 7 cm; weight: 71 ± 10 kg)	Stryd™ (Stryd Powermeter, Stryd Inc. Boulder CO, USA); RunScribe™ (Scribe Lab. Inc. San Francisco CA, USA)	Stryd™: 3D gyroscope; 3D accelerometer RunScribe™: 3D gyroscope; 3D accelerometer; 3D magnetometer	2	Lace shoe of the right leg	RunScribe™: 500 Hz	OMC (Imaging Source DFK 33UX174, The Imaging Source Europe GmbH; Germany)	Self-selected comfortable running velocity: 3.25 ± 0.36 m/s	Treadmill	Stance time, flight time, step length, step frequency
Gindre et al., 2016 [43]	20 habitual male runners (age: 31.6 ± 9.2 years, height: 178 ± 5.4 cm, weight: 72.5 ± 9.8 kg)	Myotest®	–	1	Around the waist of participants	500 Hz	The Optojump Next®; OMC (Casio High Speed EXILIM EX-FH25®, CASIO Europe GmbH, Norderstedt, Germany)	12 m/h; 15 km/h; 18 km/h; 21 km/h (60 m)	Indoor	Stance time, aerial time, step frequency
Kim et al., 2021 [44]	10 healthy male participants (age: 30.2 ± 5.3 years, height: 171 ± 15.3 cm, weight: 73.6 ± 12.4 kg	Adafruit BNO055 IMU sensors (Adafruit, New York, NY, USA)	–	2	Top of the instep of the right foot, and the right shin	100 Hz	OMC (VICON, Oxford, UK)	2.68 m/s	Indoor	Ankle dorsiflexion/plantarflexion and eversion/inversion angle
Koldenhoven and Hertel, 2018 [45]	12 recreational runners (6F, 6 M; age: 23.1 ± 5.5 years)	RunScribe™ (Scribe Labs, Inc., Half Moon Bay, CA, USA)	3D accelerometer; gyroscope	2	The back of each shoe	Hz	OMC (Vicon Motion Systems, Inc., Lake Forest, CA, USA); Instrumented treadmill (Bertec, Columbus, OH, USA)	Preferred speed: 2.7 ± 0.1 m/s; 1.5 miles	Treadmill	Stance time, cycle time, stride length, running speed
Konharn et al., 2016 [46]	30 normal-weight participants (15F, 15 M; age: 21.7 ± 1.0 years; height: 163.3 ± 19.5 cm; weight: 59.4 ± 8.5 kg)	Apps (Runtastic pedometer, Footsteps pedometer, and Walker pedometer) were downloaded into iPhone5 (iOS 7.0.3, Apple, Inc, CA, USA)	–	1	Right hip at the midline	–	The OZ1 Marathon treadmill (Marathon (Thailand) Co., Ltd., Bangkok, Thailand); The HJ-203 Omron pedometer (Omron Healthcare, Co., Ltd., Kyoto, Japan)	Moderate: 6.4 km/h; vigorous: 8 km/h	Treadmill	Running speed
Koska et al., 2018 [47]	51 recreational runners (15F, 36 M; 33.9 ± 8.2 years, height: 177.9 ± 7.6 cm; weight: 70.9 ± 10.1 kg)	IMU (aims®, Xybermind, Tübingen, Germany)	3D accelerometer (± 16 g); gyroscope (± 2000°/s)	1	The heel cup of the right shoe	400 Hz	OMC (Qualisys, Gothenburg, Sweden)	10 m/h; 12 km/h; 15 km/h	Treadmill	Rearfoot sagittal/eversion ROM
Li et al., 2020 [48]	10 healthy subjects (age > 18 years; height: 170 ± 10 cm; weight: 75 ± 10 kg)	Microelectromechanical system (MEMS) IMUs	-	7	The left side of the waist, bilateral knees above and below, bilateral feet	50 Hz	NDI system (NDI, Ontario, Canada)	5.1 km/h	Indoor	Hip, knee, and ankle angles in the sagittal plane
Mavor et al., 2020 [12]	20 civilian participants (10F, 10 M; age: 23.7 ± 3.44 years; height: 175 ± 7.93 cm; 71.9 ± 13.2 kg)	IMU (MVN BIOMECH, Xsens, Enschede, the Netherlands)	–	17	The back of the head, sacrum, sternum, and bilaterally on the upper arms, forearms, hands, shoulders, thighs, shanks, and feet	240 Hz	OMC (Vantage 5, Vicon, Oxford, UK)	-	Indoor	Hip, knee, and ankle flexion–extension, ab/adduction and axial rotation angle
Mo and Chow, 2018 [49]	11 healthy volunteers (4F, 7 M; age: 25.5 ± 4.2 years; height: 168.3 ± 9.1 cm; weight: 58.8 ± 5.3 kg)	IMU system (MyoMOTION MR3, Noraxon, USA)	3D accelerometer (± 16 g)	5	Sacrum (L5-S1), shanks (anteromedial distal aspect of the tibia) and feet (the dorsal surface of the shoe)	200 Hz	Force-platforms (Bertec, FP4060-07, USA)	Jog (3.1 ± 0.1 m/s); run (4.1 ± 1.2 m/s)	Indoor: 10 m walkway	Stance time
Nüesch et al., 2017 [50]	20 healthy subjects (12F, 8 M; age: 27.4 ± 8.3 years; height: 175 ± 8 cm; weight: 66.5 ± 12.5 kg)	IMU (RehaGait®, Hasomed, Magdeburg, Germany)	3D accelerometer (± 16 g); 3D gyroscope (± 2000°/s); 3D magnetometer (± 1.3 Gs)	7	The sacrum and bilaterally on the lateral thigh (middle), lateral shank (lower third), and lateral foot (on the shoe, below lateral malleolus)	400 Hz	OMC (Vicon MX, Vicon Motion Systems Ltd., Oxford, UK)	Self-selected running speed (2.93 ± 0.35 m/s)	Treadmill	Hip, knee, and ankle angles in the sagittal plane/at initial contact; maximal/minimal ankle angle; ankle dorsiflexion/ plantarflexion ROM; hip and knee ROM (first and second half stride)
Schmidt et al., 2016 [51]	12 track and field athletes (2F, 10 M)	IMU (MPU-9150) from InvenSense	3D accelerometer (16 bit and ± 16 g range); 3D gyroscope (16 bit and ± 1000°/s); 3D magnet field sensor	2	Ankles	1000 Hz	OptojumpNext photocell system (Microgate, Bolzano, Italy / OJ)	Maximal sprints on a 60 m track	Track	Stance time
Watari et al., 2016 [52]	22 semi-elite runners (8F, 14 M; age: 28.2 ± 10.1 years; height: 173 ± 75 cm; weight: 65.4 ± 8.1 kg)	Built-in accelerometer (Forerunner 620, Garmin International Inc., Olathe, KS)	–	1	Torso of the runner, near the xiphoid process of the sternum	–	Instrumented treadmill (Bertec, Columbus, OH); OMC (Vicon Motion System, Vicon MX3, Oxford, UK) motion capture system	2.7 m/s; 3.0 m/s; 3.3 m/s; 3.6 m/s; 3.9 m/s	Treadmill	Stance time
Wouda et al., 2018 [53]	8 healthy experienced male runners (age: 25.1 ± 5.2 years; height: 183.7 ± 4.5 cm; weight: 77.7 ± 9.4 kg)	Xsens MVN Link inertial motion capture system (Xsens, Enschede, the Netherlands)	–	17	Both shoulders, upper arms, lower arms, hands, upper legs, lower legs, feet, head, sternum, and pelvis	240 Hz	OMC (Nexus 1.8.5, Vicon, Oxford, UK); S-Mill instrumented treadmill (ForceLink, Culemborg, the Netherlands)	10 m/h; 12 km/h; 14 km/h	Treadmill	Maximum knee flexion/extension angle during Stance
Zrenner et al., 2018 [54]	27 amateur runners (6F, 21 M; age: 24.9 ± 2.4 years; height: 178.6 ± 8.0 cm)	miPod IMU sensor	Accelerometer (± 16 g); gyroscope (± 2000 ◦/s)	2	A cavity in the right and left shoes midsole	200 Hz	OMC (Vicon Motion Systems Inc., Oxford, UK)	2–6 m/s	Indoor	Stride length, running speed
Zrenner et al., 2020 [55]	29 amateur runners (6F, 23 M; age: 24.9 ± 2.4 years)	miPod IMU sensors	Accelerometer (± 16 g); gyroscope (± 2000 ◦/s)	8	A cavity in the sole of the running shoe, laterally under the ankle, at the heel, and on the instep	200 Hz	OMC (Vicon Motion Systems Inc., Oxford, UK)	2–6 m/s	Indoor	Stance time, stride time, stride length, running speed, rearfoot ROM in the frontal plane

*F* female, *M* male; *IMU* inertial measurement unit, *OMC* optical motion capture system, *ROM* range of motion

### Risk of Bias of the Included Studies

No articles were rated as HQ or VLQ, 11 as MQ and 14 as LQ (Table 4). Agreement between both assessors was good (Cohen’s kappa = 0.75; 95% CI = 0.68–0.82). The items for which articles generally scored higher were ‘1- Background and research question’, ‘4- Study design’, and ‘12- Conclusion’. By contrast, only two studies (8%) provided justification about their sample sizes.

**Table 4:** Quality assessment scoring of 25 included studies

Author(s), Year [Reference No.]	Q1	Q2	Q3	Q4	Q5	Q6	Q7	Q8	Q9	Q10	Q11	Q12	Total	%	Quality
Ammann et al., 2016 [32]	2	2	1	2	0	N	1	2	1	2	2	2	17/24	70.8%	MQ
Bergamini et al., 2012 [33]	2	1	0	2	1	N	1	2	1	1	1	1	13/24	54.2%	LQ
Brahms et al., 2018 [34]	2	2	2	2	1	N	1	2	1	2	2	2	19/24	79.2%	MQ
Cooper et al., 2009 [35]	2	1	1	2	0	N	2	2	1	0	1	1	13/24	54.2%	LQ
Day et al., 2021 [36]	1	2	0	2	1	N	1	2	1	1	1	2	14/24	58.3%	LQ
Deflandre et al., 2018 [37]	2	2	2	2	1	0	1	1	2	1	2	2	18/24	75.0%	MQ
De Fontenay et al., 2020 [38]	2	2	2	2	1	2	1	0	2	2	1	2	19/24	79.2%	MQ
Dorschky et al., 2019 [39]	2	1	0	2	1	N	2	2	1	1	1	2	15/24	62.5%	LQ
Falbriard et al., 2018 [40]	2	2	0	2	1	1	2	2	2	1	1	2	18/24	75.0%	MQ
Fox et al., 2019 [41]	2	1	0	2	2	N	1	1	1	2	2	2	16/24	66.7%	LQ
García-Pinillos et al., 2019 [42]	2	2	1	2	1	N	1	1	1	2	2	2	17/24	70.8%	MQ
Gindre et al., 2016 [43]	2	2	2	2	1	N	1	2	2	1	1	2	18/24	75.0%	MQ
Kim et al., 2021 [44]	2	2	0	2	1	N	2	2	2	1	1	2	17/24	70.8%	MQ
Koldenhoven and Hertel, 2018 [45]	1	1	2	2	1	N	1	2	1	2	1	2	16/24	66.7%	LQ
Konharn et al., 2016 [46]	2	2	0	2	1	N	1	1	1	1	2	2	15/24	62.5%	LQ
Koska et al., 2018 [47]	2	1	0	2	1	N	1	2	1	2	1	2	15/24	62.5%	LQ
Li et al., 2020 [48]	2	1	0	2	1	N	2	1	2	2	1	2	16/24	66.7%	LQ
Mavor et al., 2020 [12]	2	1	0	2	1	2	2	2	2	0	1	2	17/24	70.8%	MQ
Mo and Chow, 2018 [49]	2	0	0	2	0	N	2	2	1	1	1	2	13/24	54.2%	LQ
Nüesch et al., 2017 [50]	2	2	2	2	1	N	2	1	2	2	2	2	20/24	83.3%	MQ
Schmidt et al., 2016 [51]	1	0	0	2	1	N	1	1	1	2	1	2	12/24	50.0%	LQ
Watari et al., 2016 [52]	1	2	0	2	2	N	1	1	1	2	1	2	15/24	62.5%	LQ
Wouda et al., 2018 [53]	2	2	0	2	0	N	1	2	2	2	1	2	16/24	66.7%	LQ
Zrenner et al., 2018 [54]	2	1	0	2	1	N	1	2	2	2	2	2	17/24	70.8%	MQ
Zrenner et al., 2020 [55]	2	0	0	2	1	N	1	2	2	1	1	2	14/24	58.3%	LQ

*N* not mentioned, *MQ* moderate quality, *LQ* low quality

### Synthesis of Results

#### Validity

**Fig. 2:**
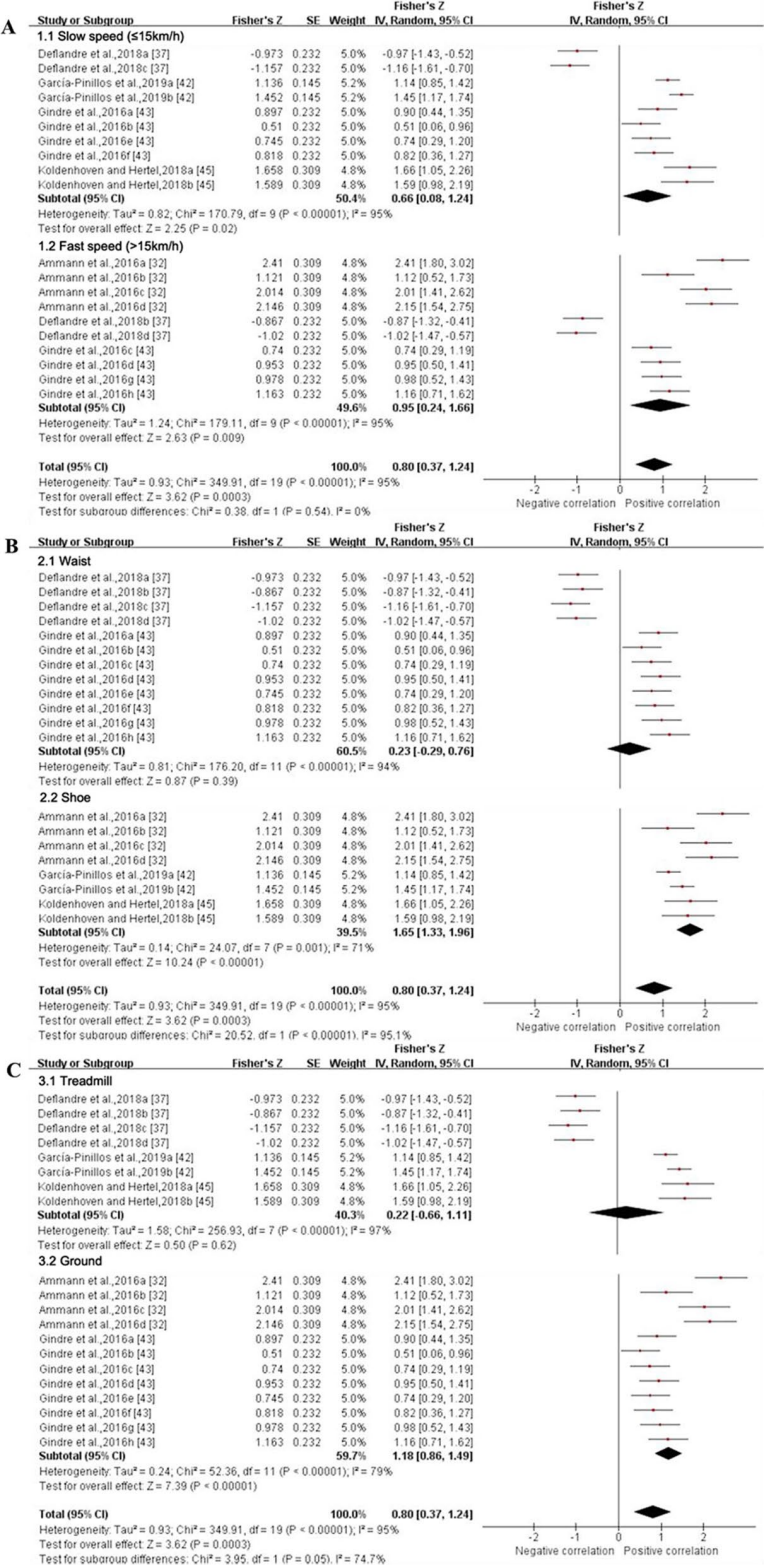
Subgroup analysis describing the validity of stance time measured using IMU (Intraclass correlation coefficient, ICC). A based on running speed, B based on location and C based on running surface. Squares represent Fisher’s Z; bars indicate 95% confidence intervals and diamonds as pooled data. Ammann et al. 2016a (combined speeds), 2016b (maximal sprinting speed), 2016c (intense training speed), 2016d (normal training speed) [32]; Deflandre et al. 2018a (8 km/h, IMUs vs optical motion capture system), 2018b (16 km/h, IMUs vs optical motion capture system), 2018c (8 km/h, IMUs vs Optogait), 2018d (16 km/h, IMUs vs Optogait) [37]; García-Pinillos et al.,2019a (IMUs vs optical motion capture system, IMUs: Stryd™), 2019b (IMUs vs optical motion capture system, IMUs: RunScribe™) [42]; Gindre et al., 2016a (12 km/h, IMUs vs optical motion capture system), 2016b (15 km/h, IMUs vs optical motion capture system), 2016c (18 km/h, IMUs vs optical motion capture system), 2016d (21 km/h, IMUs vs optical motion capture system), 2016e (12 km/h, IMUs vs Optojump), 2016f (15 km/h, IMUs vs Optojump), 2016 g (18 km/h, IMUs vs Optojump), 2016 h (21 km/h, IMUs vs Optojump) [43]; Koldenhoven and Hertel, 2018a (left limb), 2018b (right limb) [45]. *SE* standard error, *IV* inverse variance, *CI* confidence interval

**Fig. 3:**
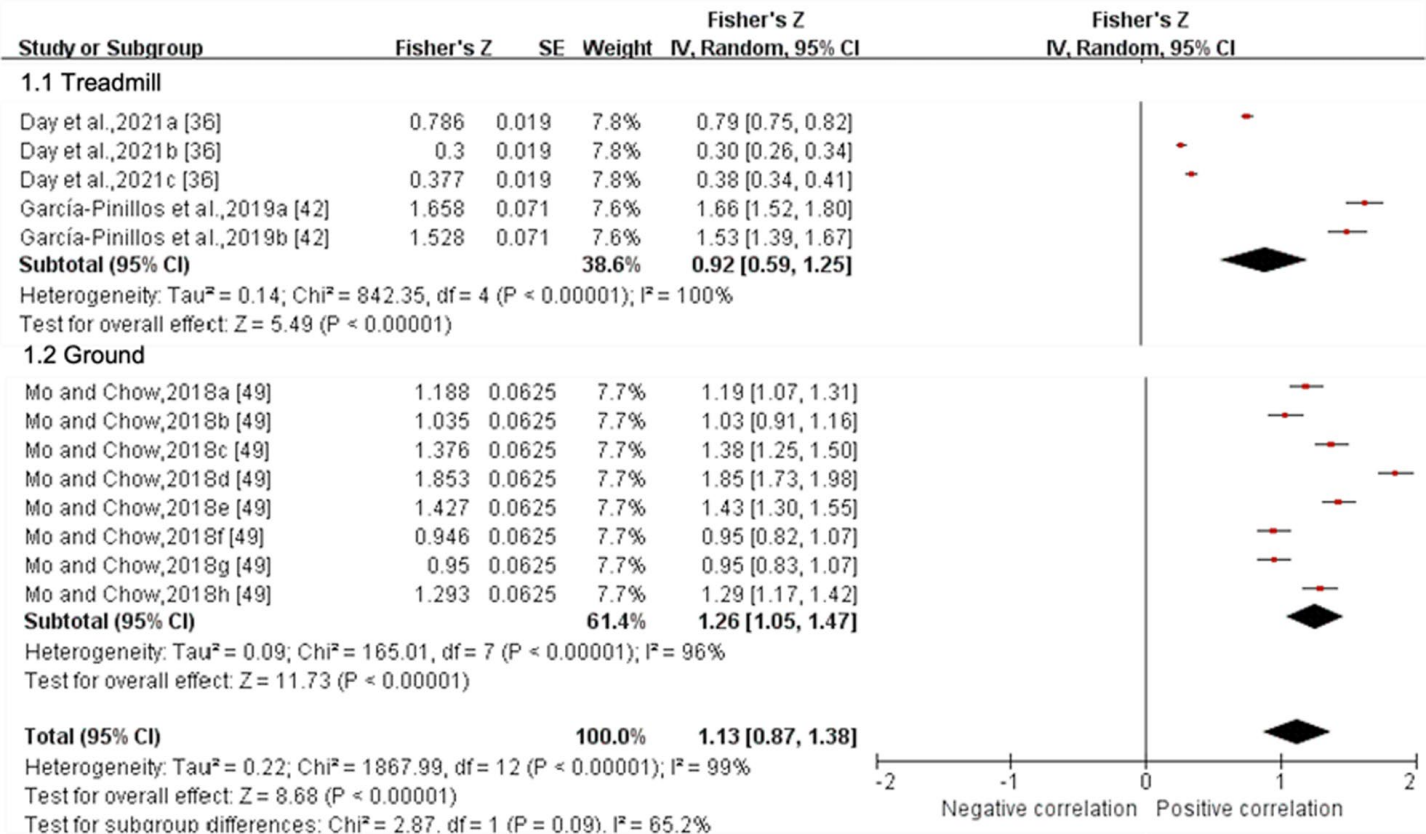
Subgroup analysis describing the validity of stance time measured using IMU (Pearson correlation coefficient, *r*). Squares represent Fisher’s Z; bars indicate 95% confidence intervals and diamonds as pooled data. Day et al.,2021a (5 Hz cutoff), 2021b (10 Hz cutoff), 2021c (30 Hz cutoff) [36]; García-Pinillos et al.,2019a (IMUs vs optical motion capture system, IMUs: Stryd™), 2019b (IMUs vs optical motion capture system, IMUs: RunScribe™) [42]; Mo and Chow, 2018a (jogging, L-method), 2018b (jogging, M-method), 2018c (jogging, S-method), 2018d (jogging, MS-method), 2018e (running, L-method), 2018f (running, M-method), 2018 g (running, S-method), 2018 h (running, MS-method) [49]. *SE* standard error, *IV* inverse variance, *CI* confidence interval.

**Fig. 4:**
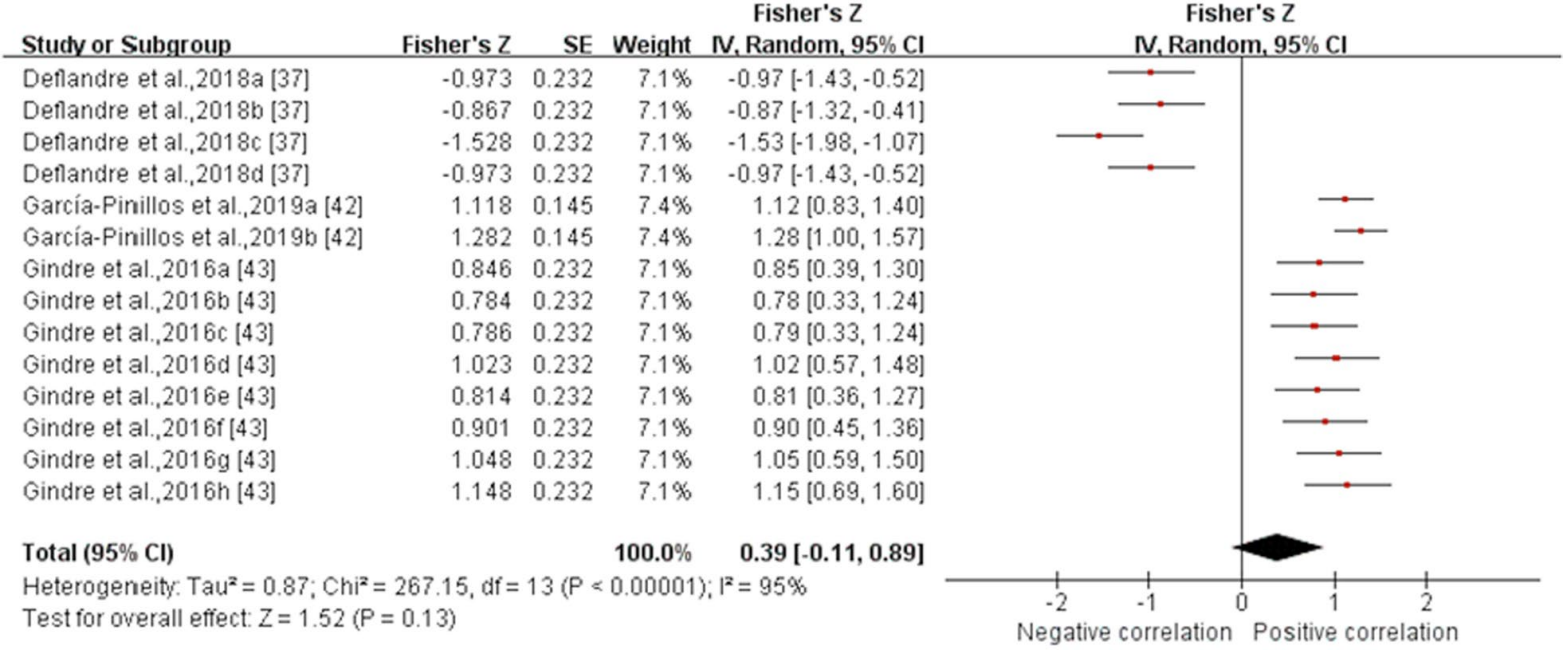
Forest plot describing the validity of flight time measured using IMU (Intraclass correlation coefficient, ICC). Squares represent Fisher’s Z; bars indicate 95% confidence intervals and diamonds as pooled data. Deflandre et al.,2018a (8 km/h, IMUs vs optical motion capture system), 2018b (16 km/h, IMUs vs optical motion capture system), 2018c (8 km/h, IMUs vs Optogait), 2018d (16 km/h, IMUs vs Optogait) [37]; García-Pinillos et al.,2019a (IMUs vs optical motion capture system, IMUs: Stryd™), 2019b (IMUs vs optical motion capture system, IMUs: RunScribe™) [42]; Gindre et al.,2016a (12 km/h, IMUs vs optical motion capture system), 2016b (15 km/h, IMUs vs optical motion capture system), 2016c (18 km/h, IMUs vs optical motion capture system), 2016d (21 km/h, IMUs vs optical motion capture system), 2016e (12 km/h, IMUs vs Optojump), 2016f (15 km/h, IMUs vs Optojump), 2016 g (18 km/h, IMUs vs Optojump), 2016 h (21 km/h, IMUs vs Optojump) [43]. *SE* standard erroSr, *IV* inverse variance, *CI* confidence interval.

**Fig. 5:**
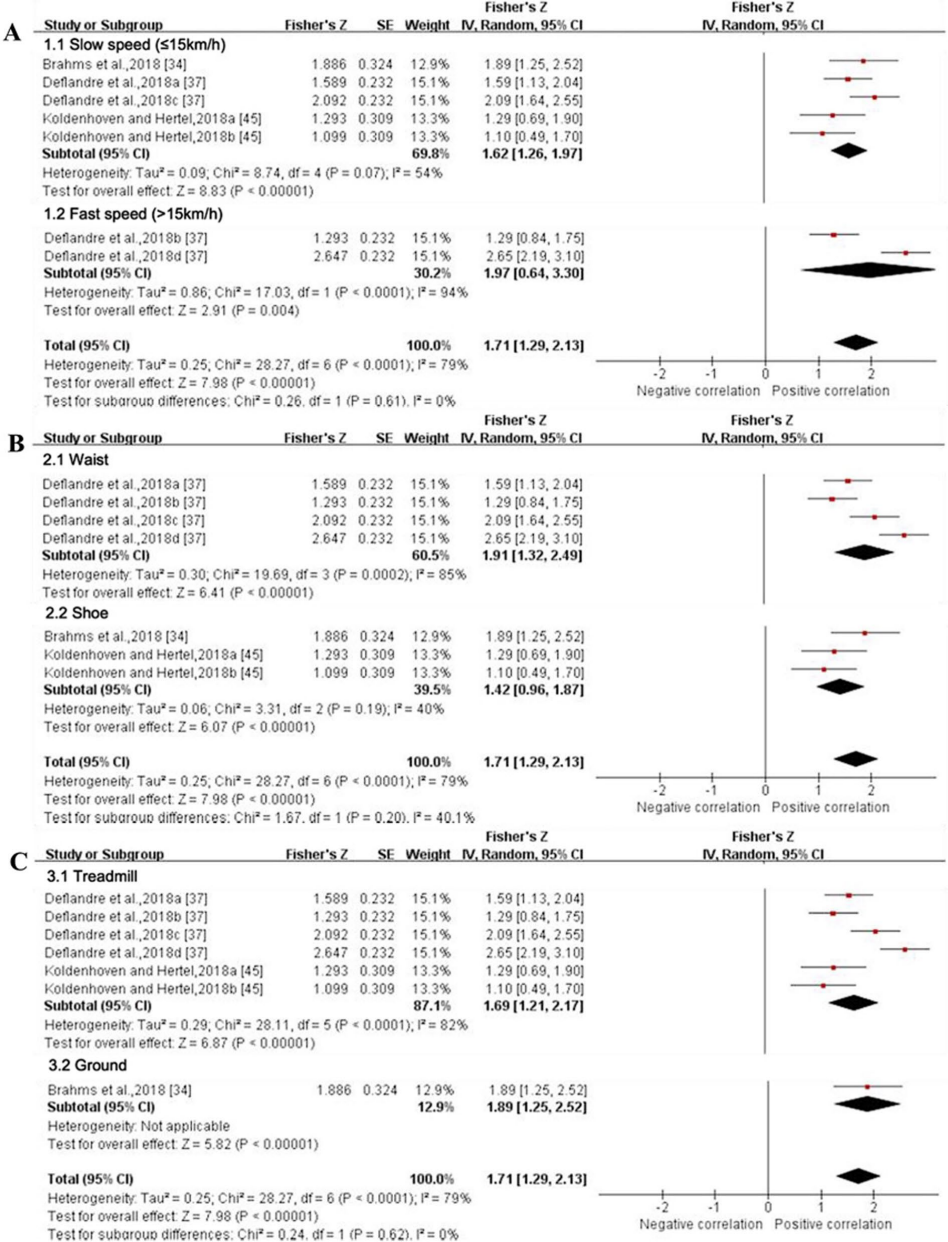
Subgroup analysis describing the validity of stride length measured using IMU (Intraclass correlation coefficient, ICC). A based on running speed, B based on location and C based on running surface. Squares represent Fisher’s Z; bars indicate 95% confidence intervals and diamonds as pooled data. Deflandre et al. 2018a (8 km/h, IMUs vs optical motion capture system), 2018b (16 km/h, IMUs vs optical motion capture system), 2018c (8 km/h, IMUs vs Optogait), 2018d (16 km/h, IMUs vs Optogait) [37]; Koldenhoven and Hertel, 2018a (left limb), 2018b (right limb) [45]. *SE* standard error, *IV* inverse variance, *CI* confidence interval.

**Fig. 6:**
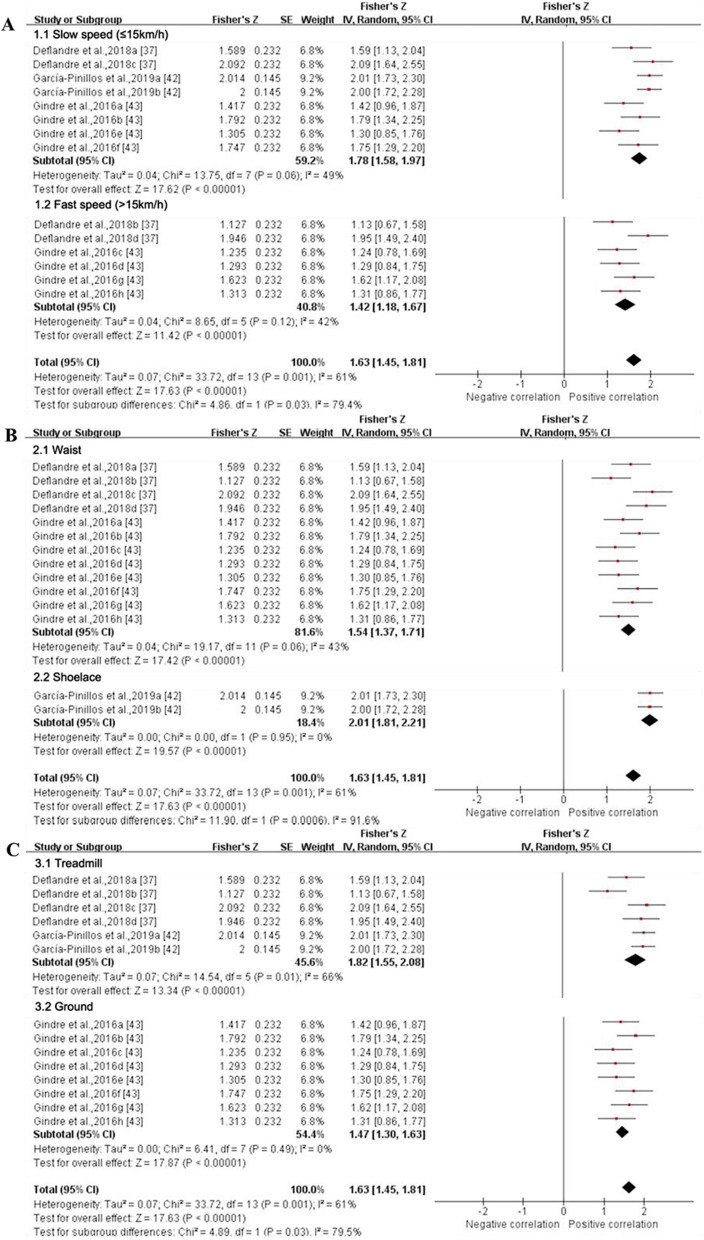
Subgroup analysis describing the validity of step frequency measured using IMU (Intraclass correlation coefficient, ICC). A based on running speed, B based on location and C based on running surface. Squares represent Fisher’s Z; bars indicate 95% confidence intervals and diamonds as pooled data. Deflandre et al. 2018a (8 km/h, IMUs vs optical motion capture system), 2018b (16 km/h, IMUs vs optical motion capture system), 2018c (8 km/h, IMUs vs Optogait), 2018d (16 km/h, IMUs vs Optogait) [37]; García-Pinillos et al.,2019a (IMUs vs optical motion capture system, IMUs: Stryd™), 2019b (IMUs vs optical motion capture system, IMUs: RunScribe™) [42]; Gindre et al.,2016a (12 km/h, IMUs vs optical motion capture system), 2016b (15 km/h, IMUs vs optical motion capture system), 2016c (18 km/h, IMUs vs optical motion capture system), 2016d (21 km/h, IMUs vs optical motion capture system), 2016e (12 km/h, IMUs vs Optojump), 2016f (15 km/h, IMUs vs Optojump), 2016 g (18 km/h, IMUs vs Optojump), 2016 h (21 km/h, IMUs vs Optojump) [43]. *SE* standard error, *IV* inverse variance, *CI* confidence interval

**Fig. 7:**
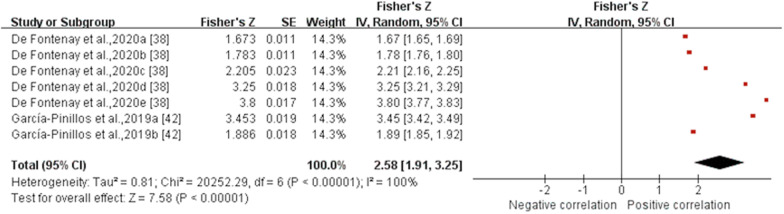
Forest plot describing the validity of step frequency measured using IMU (Pearson correlation coefficient, *r*). Squares represent Fisher’s Z; bars indicate 95% confidence intervals and diamonds as pooled data. De Fontenay et al.,2020a (IMUs vs optical motion capture system, IMUs: Moov Now™), 2020b (IMUs vs optical motion capture system, IMUs: MilestonePod), 2020c (IMUs vs optical motion capture system, IMUs: RunScribe™), 2020d (IMUs vs optical motion capture system, IMUs: Zoi), 2020e (IMUs vs optical motion capture system, IMUs: TgForce) [38]; García-Pinillos et al.,2019a (IMUs vs optical motion capture system, IMUs: Stryd™), 2019b (IMUs vs optical motion capture system, IMUs: RunScribe™) [42]. *SE* standard error, *IV* inverse variance, *CI* confidence interval

**Fig. 8:**
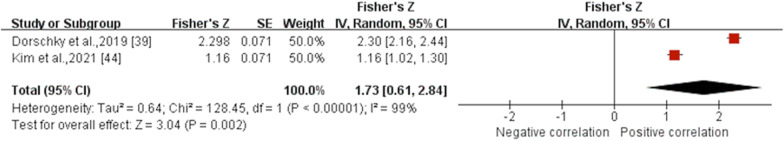
Forest plot describing the validity of running speed measured using IMU (Intraclass correlation coefficient, ICC). Squares represent Fisher’s Z; bars indicate 95% confidence intervals and diamonds as pooled data. Koldenhoven and Hertel, 2018a (left limb), 2018b (right limb) [45]; Konham et al., 2016a (moderate), 2016b (vigorous) [46]. *SE* standard error, *IV* inverse variance, *CI* confidence interval

Validity was assessed using optical motion capture system (*n* = 18), instrumented treadmill (*n* = 7), force plate (*n* = 3), timing light system (*n* = 1) and photocell system (*n* = 3) as criteria. Overall, nine gait spatiotemporal and 31 lower extremity joint kinematics parameters were assessed across the 25 studies that examined the validity of IMUs. From these outcomes, one joint kinematics and five gait spatiotemporal parameters presented sufficient study quality and statistical outcomes for data pooling (Figs. 2, 3, 4, 5, 6, 7, 8). Meta-analysis was not possible on other outcomes because of the limited number of studies or the lack of consistency in data reporting, as many studies reported only RMSE or bias. Studies that were not included in the meta-analysis were qualitatively summarised according to outcomes in Additional File 3.

##### Quantitative Pooling for Validity

*Stance time* Data from four MQ and one LQ studies suggested that the validity for stance time derived from IMUs, as reported by ICCs, was moderate (ICC (95% CI) = 0.664 (0.354, 0.845), I^2^ = 95%, *p* = 0.0003) (Fig. 2) [32, 37, 42, 43, 45], but pooled *r* values from one MQ and two LQ studies indicated validity for stance time from IMUs was excellent (*r* (95% CI) = 0.811 (0.701, 0.881), I^2^ = 99%, *p* < 0.001) (Fig. 3) [36, 42, 49]. The validity of stance time reported by *r* values can only be analysed for subgroups based on running surface due to variable running speed and the involvement of multiple attachment locations of IMUs. Subgroup analysis showed no significant effect of running speed on the validity for stance time derived from IMUs (*p* = 0.54), while IMUs at the shoe (ICC (95% CI) = 0.929 (0.869, 0.961), I^2^ = 71%) showed higher agreement compared to at the waist (ICC (95% CI) = 0.226 (− 0.282, 0.641), I^2^ = 94%) (*p* < 0.001) (Fig. 2). The validity reported via ICC and r values did not differ significantly between the two running surfaces (*p* ≥ 0.05) (Figs. 2 and 3). Sensitivity analysis showed that the results were stable.

*Flight time* Data from three MQ studies suggested that the validity for flight time measured by IMUs was poor with no statistical significance (ICC (95% CI) = 0.371 (− 0.110, 0.711), I^2^ = 95%, *p* = 0.13) (Fig. 4) [37, 42, 43]. Subgroup analysis was not conducted as the results were not statistically significant. The sensitivity analysis showed that after excluding the study of Deflandre et al. [37], the I^2^ reduced (I^2^ = 0%), summary ICC value increased (ICC (95% CI) = 0.774 (0.716, 0.818), *p* < 0.001). Sensitivity analysis showed that the results were unstable.

*Stride length* Data from two MQ and one LQ study suggested that the validity for stride length derived from IMUs was excellent (ICC (95% CI) = 0.937 (0.859, 0.972), I^2^ = 79%, *p* < 0.001) (Fig. 5) [34, 37, 45]. The results of the subgroup analysis based on running speed, IMUs’ position and running surface were not statistically significant (*p* ≥ 0.2) (Fig. 5). Sensitivity analysis showed that after excluding the study of Deflandre et al. [37] the I^2^ reduced (I^2^ = 40%), and the agreement was good (ICC (95% CI) = 0.890 (0.744, 0.954), *p* < 0.001). Sensitivity analysis showed that the results were stable.

*Step frequency* Data from four MQ studies suggested that the validity for step frequency derived from IMUs was excellent [(ICC (95% CI) = 0.926 (0.896, 0.948), I^2^ = 61%, *p* < 0.001) (Fig. 6) [37, 42, 43] and (*r* (95% CI) = 0.989 (0.957, 0.997), I^2^ = 100%, *p* < 0.001) (Fig. 7) [38, 42]]. The results of subgroup analysis based on running speed showed that the summary ICC value at fast speed (ICC (95% CI) = 0.890 (0.827, 0.932), I^2^ = 49%) was lower than that at slow speed (ICC (95% CI) = 0.945 (0.919, 0.962), I^2^ = 42%) (Fig. 6). The IMUs at the waist showed good to excellent agreement (ICC (95% CI) = 0.912 (0.879, 0.937), I^2^ = 43%), and the shoelace showed excellent agreement (ICC (95% CI) = 0.965 (0.948, 0.976), I^2^ = 0%) (Fig. 6). Running on the treadmill (ICC (95% CI) = 0.949 (0.914, 0.969), I^2^ = 66%) showed higher consistency compared to the ground (ICC (95% CI) = 0.900 (0.862, 0.926), I^2^ = 0%) (Fig. 6). Due to the limited amount of literature, no subgroup analysis was performed on the validity of the step frequency measured by IMUs as reported through the *r* values. Sensitivity analysis showed that the results were stable.

*Running speed* Data from two LQ studies suggested that the validity for running speed measured by IMUs was good (ICC (95% CI) = 0.848 (0.523, 0.958), I^2^ = 88%, *p* = 0.0003) (Fig. 8) [45, 46]. Subgroup analyses could not be performed due to the insufficient number of studies. Sensitivity analysis showed that the results were stable.

**Fig. 9:**
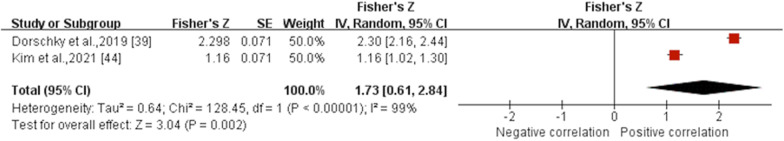
Forest plot describing the validity of ankle angle in the sagittal plane measured using IMU (Pearson correlation coefficient, *r*). Squares represent Fisher’s Z; bars indicate 95% confidence intervals and diamonds as pooled data. *SE* standard error, *IV* inverse variance, *CI* confidence interval

*Ankle angle in the sagittal plane* Data from one MQ and one LQ study suggested that the validity for ankle angle in the sagittal plane measured by IMUs was excellent (*r* (95% CI) = 0.939 (0.544, 0.993), I^2^ = 99%, *p* = 0.002) (Fig. 9) [39, 44]. Subgroup and sensitivity analyses could not be performed due to the insufficient number of studies.

#### Reliability

**Fig. 10:**
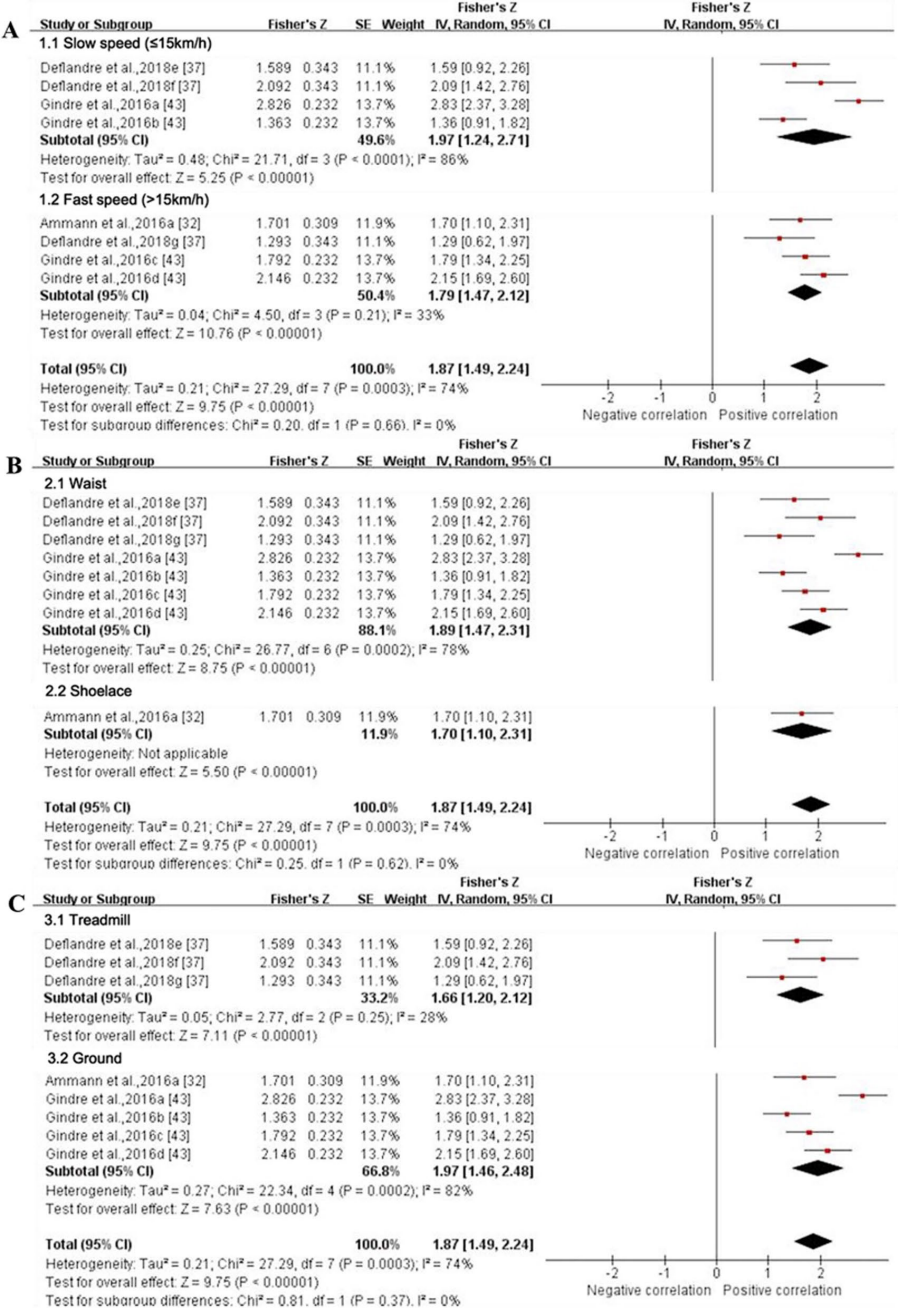
Subgroup analysis describing the reliability of stance time measured using IMUs. A based on running speed, B based on location and C based on running surface. Squares represent Fisher’s Z; bars indicate 95% confidence intervals and diamonds as pooled data. Deflandre et al.,2018e (8 km/h), 2018f (12 km/h), 2018 g (16 km/h) [37]; Gindre et al.,2016a (12 km/h), 2016b (15 km/h), 2016c (18 km/h), 2016d (21 km/h) [43]. *SE* standard error, *IV* inverse variance, *CI* confidence interval

**Fig. 11:**
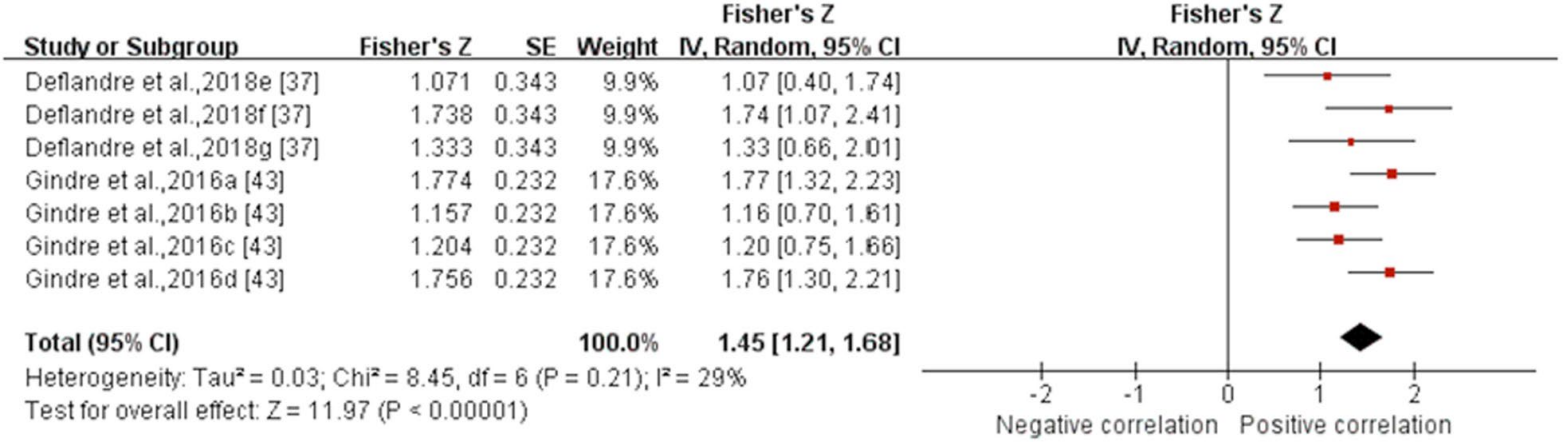
Forest plot describing the reliability of step frequency measured using IMUs. Squares represent Fisher’s Z; bars indicate 95% confidence intervals and diamonds as pooled data. Deflandre et al., 2018e (8 km/h), 2018f (12 km/h), 2018 g (16 km/h) [37]; Gindre et al., 2016a (12 km/h), 2016b (15 km/h), 2016c (18 km/h), 2016d (21 km/h) [43]. *SE* standard error, *IV* inverse variance, *CI* confidence interval.

Six gait spatiotemporal outcomes and 22 lower extremity joint kinematics outcomes were assessed across the six studies that examined reliability for IMUs. From this group, only two gait spatiotemporal outcomes presented sufficient study quality and statistical outcomes for meta-analysis (Figs. 10 and 11). Similar to validity, the inability to pool outcomes were due to either a limited number of studies or a lack of consistency in data reporting. Studies that were unable to be pooled were qualitatively summarised according to outcomes in Additional File 3.

##### Quantitative Pooling for Reliability

*Stance time* Data from three MQ studies suggested that the reliability for stance time measured by IMUs was excellent (ICC (95% CI) = 0.954 (0.903, 0.978), I^2^ = 74%, *p* < 0.001) (Fig. 10) [32, 37, 43]. Subgroup analysis showed no significant effect of running speed, IMU’s position and running surface on the reliability for stance time derived from IMUs (*p* ≥ 0.37). Sensitivity analysis showed that the results were stable.

*Step frequency* Data from two MQ studies suggested that the reliability for flight time measured by IMUs was good (ICC (95% CI) = 0.896 (0.837, 0.933), I^2^ = 29%, *p* < 0.001) (Fig. 11) [37, 43]. Subgroup analyses could not be performed due to the insufficient number of studies. Sensitivity analysis showed that the results were stable.

## Discussion

The aim of this review was to determine the concurrent validity and test–retest reliability of biomechanical outcomes derived from IMUs during running in healthy adults. The main findings of this review were as follows: (1) among the studies examining the validity or reliability of measurements from IMUs during running, there have been noticeably more studies involving gait spatiotemporal outcomes than those involving lower limb joint kinematics. (2) Regarding validity: the stride length, step frequency and ankle angle in the sagittal plane showed excellent agreement, the stance time depicted moderate to excellent agreement and running speed was good, with statistical significance (*p* < 0.01), whereas the summary Fisher's Z value of flight time was not statistically significant (*p* = 0.13). (3) For reliability: stance time showed excellent test–retest reliability and step frequency showed good test–retest reliability, and summary Fisher’s Z values were statistically significant (*p* < 0.001).

This systematic review used a similar review process to the previous study, which evaluated the validity and reliability of measurements from IMUs during walking [17]. As far as the authors know, this is the first meta-analysis involving the assessment of validity and reliability of lower limb joint kinematics measured by IMUs during running. It has been reported that running speed, IMUs’ position and running surface are the main factors related to the validity and reliability of measurements from IMUs [41, 56]. Therefore, to explore the specific effects of these factors on the validity and reliability of gait spatiotemporal outcomes and lower extremity joint kinematics derived from IMUs, the subgroup analyses based on running speed, IMUs’ position and running surface were conducted for the parameters that could be pooled.

In this systematic review and meta-analysis, the included studies have measured the validity and reliability of IMUs’ measurements at a variety of running speeds (7.2–21 km/h). Specifically, two studies measured gait spatiotemporal parameters and sagittal joint kinematics at preferred running speed [42, 50]. Although difference in preferred running speed was found between them (2.93 ± 0.35 m/s and 3.25 ± 0.36 m/s), both studies suggested that the measurements from IMUs and optical motion capture systems had an almost perfect association (ICC > 0.81 and CMC > 0.950) [42, 50]. In addition, five studies assessed running at maximum speed, and a general conclusion was that speed had an impact on the validity of the measurements from IMUs [32, 33, 40, 41, 51]. Although only the validity of step frequency derived from IMUs was statistically different in the subgroup analysis based on running speed (*p* = 0.03), a summary of other statistical outcomes in Additional File 3 also showed a general decrease in the validity and reliability of gait spatiotemporal parameters and lower limb joint kinematics as running speed increased [32, 37, 41, 43, 49, 52, 56]. This can be explained by the fact that increase in running speed increases peak vertical acceleration at impact, resulting in soft tissue artifacts [57, 58]. However, the results of Watari et al. [52] showed that the validity is the lowest at the lowest speed (2.7 m/s), similar to the results of Gindre et al. [43]. Difference in IMUs’ placement may be one of the main reasons for the discrepancies in the conclusion [58, 59].

Collectively, foot and shank were the most common IMUs attachment locations when assessing the validity or reliability of IMUs measurement of running kinematics, which is consistent with previous studies [40, 49, 60]. Subgroup analysis based on IMU’s position depicted that for stance time and step frequency, placing the IMUs on the shoe yielded more accurate measurements than placing the IMUs around the waist (*p* < 0.001). Previous studies generally suggested that IMUs’ position closer to the foot can more accurately capture acceleration signals and thus recognise gait events [40, 49, 60]. However, a recent review showed that placing the IMUs on the foot, tibia and lumbar spine yielded valid and reliable stride data, suggesting measurement position may not be a critical factor [18]. Since only subgroup analyses on the waist and shoe of two gait parameters were performed, it is therefore unclear whether other IMUs’ placements affect the gait spatiotemporal and lower extremity kinematics outcomes.

In this review, running on an indoor track or walkway, running on a treadmill and running outside were included. Previous evidence suggests that running on a treadmill and running on the ground/track are associated with different biomechanical performance [56, 61–63], so we divided the running surfaces into treadmill and ground before carrying out subgroup analyses. The results of the subgroup analysis based on running surface showed that the validity of running on a treadmill was better than running on the ground for step frequency. Interestingly, close to half of the studies in our review were conducted on a treadmill [35–38, 40, 42, 45–47, 50, 52, 53]. The effective control of running speed on treadmills is the main reason. On the other hand, it also provides support for researchers to assess the effect of specific running speed on the validity and reliability of the IMUs’ measurements. Moreover, to improve ecological validity, IMUs are necessary for outdoor measurement.

Additionally, one study compared different algorithms of IMUs, and the results showed that results obtained by different algorithms vary greatly [54]. This means that the algorithm optimisation is one of the effective methods to improve the validity of measurements from IMUs. Previous studies have pointed out that the data type of IMU data and the corresponding calculation method areimportant factors in measurement errors [53, 64, 65]. IMUs included in the current review were provided by different manufacturers, it is difficult to summarize their calculation procedures, so this review cannot provide strong support for the view. One study compared five commercial IMUs: MilestonePod, Moov Now™, TgForce, Zoi and RunScribe™, although they showed excellent agreement in step frequency (*r* ≥ 0.955) [38]. For the kinetic parameters, the results vary considerably (*r* values range from − 0.532 to 0.813) [38]. The finding indicated that different IMUs do have a considerable effect on the measurements. Owing to the validity of the measurement from IMUs is affected by numerous factors, further research is needed in the future.

For homogeneity, the present systematic review and meta-analysis has only reviewed healthy adults. However, measuring subjects with RRIs not only provide evidence that supports the application of IMUs in real life but also allows the optimization of the motion assessment of different people for IMUs. By comparing the kinematic results obtained by the IMUs in healthy and injured subjects during running, the results may not only provide a better understanding of the specific biomechanical mechanisms underlying injuries but also may provide coaches or clinicians with early warning of the occurrence of RRIs. Meanwhile, Bergamini et al. [33] believed that IMUs can be used in monitoring the running of amateur and elite athletes, which was similar to the results in Schmidt et al. [51]. However, one study suggested that the use of IMUs in measuring continuous motion should be considered carefully [41]. The explanation for the contrary conclusion may be that different studies used different types of IMUs and placement. Some studies used IMUs in the continuous analysis of marathon running, and the results showed that IMUs can detect significant changes in running kinematics as mileage increased [66, 67]. These results showed the possibility of applying IMUs in the evaluation of running techniques over a long period of time in specific setting.

The included studies showed low to moderate methodological quality, with scores ranging between 12 and 20 out of 24. The lack of quality research reduces the ability to make any strong conclusions or clear recommendations in this review. Similar to previous study, the sample size was underpowered and/or unjustified in most of the literature which limits the statistical power of the available data [17]. Only two of the studies conducted an a priori power analysis for sample size [41, 52], and more than half of the included studies had a sample size of 12 or less. Furthermore, the paucity of use of appropriate statistical tests was also a prominent issue, with nearly half of the studies not reporting both absolute and relative statistical metrics, or Bland–Altman plots as a visual representation of agreement [68]. Considering these findings, more HQ studies are needed in the future.

### Validity

In this review, although all the included studies compared the measurement results derived from IMUs with the reference systems, there were few data that could be quantitatively analysed, and most of them were gait spatiotemporal parameters. Meanwhile, all pooled outcomes had moderate to high heterogeneity (I^2^ ≥ 61%). The reason for the high heterogeneity is that there were not adequate outcomes that could be subjected to meta-analysis, and most of them were from different velocity situations within the same study.

Among the gait spatiotemporal outcomes, the stance time has the most pooled data with moderate evidence and depicted moderate to excellent agreement. The reason for the relatively low agreement maybe that one of the included studies showed very poor ICCs among gait temporal outcomes, which also occurred in the flight time [37]. Considering the experimental settings and IMUs brands vary greatly among different studies, which is also an important source of heterogeneity, this study was not excluded from the meta-analysis. However, it should be interpreted with caution. Fewer studies evaluated the validity of the stride length, but still found excellent validity in all pooled data with moderate evidence, which is consistent with a previous review [17]. With regard to step frequency, the agreement was excellent and drawn from moderate evidence in the running speed range of 8 km/h to 21 km/h. As for running speed, the results showed a significant asymmetry of the left and right limbs [45], which directly led to running speed only showing good agreement in meta-analysis.

Regarding the validity of lower extremity joint kinematics, only the ankle angle in the sagittal plane can be quantified [17, 18]. It was suggested by moderate evidence that the agreement between the ankle angle in the sagittal plane obtained by the IMUs and the reference systems was excellent. One study showed that the IMUs’ measurement results in the hip, knee and ankle joints distinctly improved after offset correction, with RMSEs between 18° and 28° reduced to between 5° and 8° [50]. In addition, compared with walking, the offset between waveforms increased during running, indicating that the motion amplitude would affect the lower limb joint kinematics derived from IMUs [50]. One explanation is that increase in the amplitude of movement may reduce the accuracy of the identification of the initial angle after calibration with the IMUs [50]. For joint discrete parameters, the RMSE of the hip joint (25.1°–36.1°) was greater than that of the knee joint (13.2°–20.0°) and ankle joint (14.4°–19.1°) [50]. Moreover, one study suggested that, compared to the optical motion capture systems, lower limb RMSEs for joint angles calculated using the IMUs data were less than 10° for all axes and more rapid motions involving larger ranges of motion would probably induce greater RMSEs [12]. For rearfoot range of motion, bias increased with velocity on the sagittal plane but had no effect on eversion [47].

### Reliability

In contrast to validity, the reliability of measurements derived from IMUs during running was assessed by few studies. Similar to studies assessing validity, high quality research on reliability is lacking. Only stance time and step frequency were included in the meta-analysis. The agreement of stance time was excellent with relatively high heterogeneity (I^2^ = 74%). None of the subgroup analyses based on running speed, IMUs’ position and running surface were statistically significant (*p* ≥ 0.37) and there was excellent agreement across subgroups (summary ICC ≥ 0.930), suggesting that IMUs are robust in measuring gait temporal outcomes. Step frequency depicted good reliability with moderate heterogeneity (I^2^ = 29%), and sensitivity analysis showed that the results were stable. Only the test–retest reliability (within–tester reliability) of the measurements from IMUs was summarised because all included studies only reported the test–retest reliability, lacking studies on between–tester reliability. Among them, only one study evaluated the reliability between different days (three test sessions, with a time interval of 7 days between each test day) [37], and the remaining five studies only reported the agreement between the test and retest after a short time rest [32, 43, 44, 46, 50].

For reliability, flight time, step length, stride length, running speed and joint kinematics outcomes with the exception of ankle angle in the sagittal plane were only reported in one study, and thus evidence was limited. Furthermore, subgroup analysis based on running speed could not be performed for parameters other than stance time due to a lack of available data. However, for stance time, flight time, step length and stride length, the CV values increased with increasing running velocity [37, 43]. This finding implies that running speed affects the reliability of measurements from IMUs. RMSE did not show results similar to validity for lower extremity joint kinematics. This result indicated a high degree of consistency in the reliability of the hip, knee and ankle measurements obtained by IMUs. However, due to a limited number of studies were included in each parameter, therefore, these findings should be interpreted cautiously.

Calibration (alignment of the IMUs axes with the anatomical segment axes) enables the initial value of the IMUs to be in a prescribed standard state. It is an essential factor affecting IMU reliability, as different calibration protocols may result in substantially different measurements [69, 70]. In this review, only four studies described calibration procedures in detail, including static [48, 50] and functional movements [40, 44, 50]. Thus, it was unable to evaluate and summarise the calibration procedure, as in a previous study [71]. It is generally believed that a standardised measurement process for IMUs is necessary to the enhancement of the comparability among different studies.

### Limitations

To enhance quality control, the search was restricted to fully peer-reviewed published articles, and thus relevant conference papers may have been excluded. Only included specific gait spatiotemporal parameters and joint kinematics parameters but did not include acceleration, impact, gait events and foot strike pattern, which may lead to selection bias. In the meta-analysis, only ICC and *r* values were pooled, and the studies that illustrated ICC values without specific values were excluded, and thus the number of articles that could be pooled was reduced. Furthermore, the level of heterogeneity was substantial in most parameters. Thus, these meta-analyses should be interpreted cautiously. In addition, none of the literature in this review was rated as high quality, and thus studies that had higher quality and investigated the validity and reliability of IMUs for lower extremity kinematics during running are needed.

## Conclusion

Measuring running kinematics using IMUs helps in efficiently monitoring RRIs and evaluating running techniques in real-world settings. The findings of this review demonstrate that IMUs perform moderate to excellent correlation with gold standard for gait spatiotemporal parameters during running in healthy adults but should be reported with caution when lower extremity joint kinematics are assessed. Further, high quality literature on the validity and reliability of IMUs during running are lacking, and sample sizes seem generally underpowered. Thus, future studies should include more runners with different running skill levels and RRIs, as they may provide evidence that supports the application of IMUs in a variety of specific sports environments and provide the possibility for algorithm improvement. In addition, literature quality should be enhanced, and guidelines for the use of IMUs in running should be developed.

## Supplementary Information

**Additional file 1.** Complete search strategy.

**Additional file 2**. Complete inclusion and exclusion criteria and definition of spatiotemporal parameters.

**Additional file 3**. Qualitative summary of validity and reliability for biomechanical parameters.

## Data Availability

All data generated or analysed during this review are included in this published article and its supplementary information files.
