# Supplementary material for: Validity and Reliability of Inertial Measurement Units on Lower Extremity Kinematics During Running: A Systematic Review and Meta-Analysis
Source: Sports Med Open. 2022 Jun 27;8:86. doi: 10.1186/s40798-022-00477-0 (PMC9237201; doi:10.1186/s40798-022-00477-0)
Supplement: Supplementary file 1 — Additional file 1. Complete search strategy. [file 40798_2022_477_MOESM1_ESM.docx]

**Complete Search Strategy**

In the search strategy, a combination of terms was concluded: inertial measurement units, gait spatiotemporal and lower extremity kinematics outcomes, running and validity or reliability.

Databases: **PubMed, CINAHL, Embase, Scopus and Web of Science**

**PubMed:**

*Inertial Measurement Units:* wearable sensor* OR inertial sensor* OR inertial motion capture OR "Wearable Electronic Devices"[Mesh] OR inertial measurement unit* OR IMU OR "Micro-Electrical-Mechanical Systems"[Mesh] OR MEMS OR acceleromet* OR gyroscop* OR magnetomet* OR smart phone OR "Smartphone"[Mesh]

*Gait spatiotemporal and lower extremity kinematics outcomes:* running speed OR cadence OR (step frequency) OR (stride frequency) OR (step time) OR (stride time) OR (cycle time) OR (contact time) OR (swing time) OR (flight time) OR (step length) OR (stride length) OR spatiotemporal OR Spatio-Temporal OR "Spatio-Temporal Analysis"[Mesh] OR kinematic* OR biomechanic* OR (joint angle) OR hip OR knee OR ankle OR (range of motion) OR "Range of Motion, Articular"[Mesh]

*Running:* running OR jogging OR sprinting

*Validity or Reliability:* validity OR reliability OR feasibility OR repeatability OR consistency OR accuracy OR reproducibility OR "Reproducibility of Results"[Mesh] OR "Data Accuracy"[Mesh]

**CINAHL:**

*Inertial Measurement Units:* AB OR TI OR SU= wearable sensor* OR inertial sensor* OR inertial motion capture OR wearable electronic device* OR inertial measurement unit* OR IMU OR MEMS OR Micro-Electrical-Mechanical System* OR acceleromet* OR gyroscop* OR magnetomet* OR smart phone* OR smartphone

*Gait spatiotemporal and lower extremity kinematics outcomes:* AB OR TI OR SU= (running speed) OR cadence OR (step frequency) OR (stride frequency) OR (step time) OR (stride time) OR (cycle time) OR (contact time) OR (swing time) OR (flight time) OR (step length) OR (stride length) OR spatiotemporal OR spatio-temporal OR kinematic* OR biomechanic* OR (joint angle) OR hip OR knee OR ankle OR (range of motion)

*Running:* AB OR TI OR SU= (running OR jogging OR sprinting)

*Validity or Reliability:* AB OR TI OR SU= (validity OR reliability OR feasibility OR reproducibility OR repeatability OR consistency OR accuracy)

**Embase:**

*Inertial Measurement Units:* ‘wearable sensor*’ OR ‘inertial sensor*’ OR ‘inertial motion capture’ OR ‘wearable electronic device*’ OR ‘inertial measurement unit*’ OR IMU OR MEMS OR acceleromet* OR gyroscop* OR magnetomet* OR smartphone OR ‘smart phone’

*Gait spatiotemporal and lower extremity kinematics outcomes:* ‘running speed’ OR cadence OR ‘step frequency’ OR ‘stride frequency’ OR ‘step time’ OR ‘stride time’ OR ‘cycle time’ OR ‘contact time’ OR ‘swing time’ OR ‘flight time’ OR ‘step length’ OR ‘stride length’ OR spatiotemporal OR ‘Spatio-Temporal’ OR kinematic* OR biomechanic* OR ‘joint angle’ OR hip OR knee OR ankle OR ‘range of motion’

*Running:* running OR jogging OR sprinting

*Validity or Reliability:* validity OR reliability OR feasibility OR reproducibility OR repeatability OR consistency OR accuracy

**Scopus:**

*Inertial Measurement Units:* TITLE-ABS-KEY ((wearable AND sensor*) OR (inertial AND sensor*) OR (inertial AND motion AND capture) OR (wearable AND electronic AND device*) OR (inertial AND measurement AND unit*) OR imu OR mems OR (micro-electrical-mechanical AND system*) OR acceleromet* OR gyroscop* OR magnetomet* OR (smart AND phone) OR smartphone*) AND (LIMIT-TO ( DOCTYPE, "ar" ))

*Gait spatiotemporal and lower extremity kinematics outcomes:* TITLE-ABS-KEY ((running AND speed) OR cadence OR (step AND frequency) OR (stride AND frequency) OR (step AND time) OR (stride AND time) OR (cycle AND time) OR (contact AND time ) OR (swing AND time) OR (flight AND time) OR (step AND length) OR (stride AND length) OR spatiotemporal OR (spatio-temporal) OR kinematic* OR biomechanic* OR (joint AND angle) OR hip OR knee OR ankle OR (range AND of AND motion)) AND (LIMIT-TO (DOCTYPE, "ar" ))

*Running:* TITLE-ABS-KEY (running OR jogging OR sprinting) AND (LIMIT-TO (DOCTYPE, "ar"))

*Validity or Reliability:* TITLE-ABS-KEY (validity OR reliability OR feasibility OR reproducibility OR repeatability OR consistency OR accuracy) AND ( LIMIT-TO (DOCTYPE, "ar"))

**Web of Science:**

*Inertial Measurement Units:* TS = wearable sensor* OR inertial sensor* OR inertial motion capture OR "Wearable Electronic Devices"[Mesh] OR inertial measurement unit* OR IMU OR "Micro-Electrical-Mechanical Systems"[Mesh] OR MEMS OR acceleromet* OR gyroscop* OR magnetomet* OR "Smartphone"[Mesh]

*Gait spatiotemporal and lower extremity kinematics outcomes:* TS = running speed OR cadence OR (step frequency) OR (stride frequency) OR (step time) OR (stride time) OR (cycle time) OR (contact time) OR (swing time) OR (flight time) OR (step length) OR (stride length) OR spatiotemporal OR Spatio-Temporal OR "Spatio-Temporal Analysis"[Mesh] OR kinematic* OR biomechanic* OR (joint angle) OR hip OR knee OR ankle OR (range of motion) OR "Range of Motion, Articular"[Mesh]

*Running:* TS = (running OR jogging OR sprinting)

*Validity or Reliability:* TS = validity OR reliability OR feasibility OR repeatability OR consistency OR accuracy OR reproducibility OR "Reproducibility of Results"[Mesh] OR "Data Accuracy"[Mesh]
