# Supplementary material for: Validity and Reliability of Inertial Measurement Units on Lower Extremity Kinematics During Running: A Systematic Review and Meta-Analysis
Source: Sports Med Open. 2022 Jun 27;8:86. doi: 10.1186/s40798-022-00477-0 (PMC9237201; doi:10.1186/s40798-022-00477-0)
Supplement: Supplementary file 2 — Additional file 2. Complete inclusion and exclusion criteria and definition of spatiotemporal parameters. [file 40798_2022_477_MOESM2_ESM.docx]

**Complete Inclusion and Exclusion Criteria**

**Inclusion:**

1. evaluated the validity or reliability of inertial measurement units (IMUs),

- Concurrent validity: reported the results of simultaneous measurement by IMUs and reference systems.
- Test–retest reliability: reported the consistency between repeated measurements obtained by IMUs.

1. measured specific gait spatiotemporal and lower extremity kinematics outcomes,

- Included gait spatiotemporal outcomes: stance time, flight time, swing time, step time, stride time, step length, stride length, step frequency and running speed.
- Included lower extremity kinematics outcomes: hip, knee and ankle joints measurements.

1. compared the measurements captured by IMUs to the reference systems,

- *Reference systems*: optical motion capture system, photocell system, force plate(s), instrumented treadmill/mat and electronic timing light.

1. collected data during running, jogging or sprinting,
2. assessed healthy human beings,
3. and published in English.

**Exclusion:**

1. no relevant outcomes,

- Excluded outcomes: acceleration, symmetry and variability, impact, force, stiffness, gait events and foot strike pattern.

1. IMUs was used to measure activity or movement classification,
2. IMUs was used to evaluate energy expenditure,
3. only assessed upper limb motion,
4. no running-related activities were involved, or measurements derived from IMUs during running were reported,
5. not healthy human subjects,
6. or published in other languages.

**Definition of spatiotemporal parameters:**

| Parameter | Definition |
| --- | --- |
| Stance time | Time between initial contact to toe-off of the same foot |
| Flight time | Time between toe-off to next initial contact of the opposite foot |
| Swing time | Time between toe-off to next initial contact of the same foot |
| Step time | Time between initial contacts of the opposite foot |
| Stride time | Time between initial contacts of the same foot |
| Step length | Length or distance between initial contacts of the opposite foot |
| Stride length | Length or distance between initial contacts of the same foot |
| Step frequency | Number of initial contacts per minute |
| Running speed | The mean of stride length divided by stride time |
