# Supplementary material for: Validity and Reliability of Inertial Measurement Units on Lower Extremity Kinematics During Running: A Systematic Review and Meta-Analysis
Source: Sports Med Open. 2022 Jun 27;8:86. doi: 10.1186/s40798-022-00477-0 (PMC9237201; doi:10.1186/s40798-022-00477-0)
Supplement: Supplementary file 3 — Additional file 3. Qualitative summary of validity and reliability for biomechanical parameters. [file 40798_2022_477_MOESM3_ESM.docx]

Supplementary Table 1: Qualitative summary of validity for biomechanical parameters.

| Outcome | Study | Results | | | | Evidence |
| --- | --- | --- | --- | --- | --- | --- |
|  |  | ICC/r (95%CI) | RMSE/SEM | Bias | LoA |  |
| Stance time | Ammann et al.,2016 [32] | All speeds  ICC = 0.984 (0.977–0.989)  Maximal sprinting speed  ICC = 0.808 (0.653–0.894)  Intense training speed  ICC = 0.956 (0.921–0.976)  Normal training speed  ICC = 0.973 (0.916–0.988) | All speeds: ± 17.4 ms | All speeds: -1.9 ms  Maximal sprinting speed: 0.4 ms  Intense training speed: -0.7 ms  Normal training speed: -5.6 ms | All speeds  -19.2–15.5 ms | Moderate |
|  | Day et al.,2021 [36] | 5 Hz cutoff  r = 0.656  10 Hz cutoff  r = 0.291  30 Hz cutoff  r = 0.360 | - | - | - |  |
|  | Watari et al.,2016 [52] | 2.7 m/s: CCC = 0.69  3.0 m/s: CCC = 0.77  3.3 m/s: CCC = 0.87  3.6 m/s: CCC = 0.83  3.9 m/s: CCC = 0.84 | - | 2.7 m/s: -17.02 ms  3.0 m/s: -10.14 ms  3.3 m/s: -5.82 ms  3.6 m/s: -2.59 ms  3.9 m/s: -1.40 ms | 2.7 m/s: 43.68 ms  3.0 m/s: 45.71 ms  3.3 m/s: 43.93 ms  3.6 m/s: 38.72 ms  3.9 m/s: 44.02 ms |  |
|  | Schmidt et al.,2016 [51] | - | 4.3 ± 3.2 ms | -2.5 ± 4.8 ms | -11.8–6.8 ms |  |
|  | Mo and Chow,2018 [49] | Jogging  L-method: r = 0.830  M-method: r = 0.776  S-method: r = 0.880  MS-method: r = 0.952  Running  L-method: r = 0.891  M-method: r = 0.738  S-method: r = 0.740  MS-method: r = 0.860 | - | Jogging  L-method: 10.3 ± 8.9 ms  M-method: 38.0 ± 9.4 ms  S-method: -24.7 ± 14.8 ms  MS-method: 7.3 ± 6.2 ms  Running  L-method: 4.6 ± 12.1 ms  M-method: 32.9 ± 34.1 ms  S-method: -56.0 ± 9.6 ms  MS-method: -1.3 ± 7.1 ms |  |  |
|  | Bergamini et al.,2012 [33] | Correlation: 0.01 | - | ≈ 5 ms | <25 ms |  |
|  | Falbriard et al.,2018 [40] | - | 1–5 ms | -38–9 ms | - |  |
|  | Koldenhoven and Hertel,2018 [45] | Left limb  ICC = 0.93  Right limb  ICC = 0.92 | Left limb: SEM = 6.2 ms  Right limb: SEM = 6.4 ms | Left limb: 27.8 ms  Right limb: 29.1ms | Left limb: 9.8–45.9 ms  Right limb: 11.5–46.6 ms |  |
|  | Gindre et al.,2016 [43] | IMUs vs. Optojump  12km/h: ICC = 0.632  15km/h: ICC = 0.674  18km/h: ICC = 0.752  21km/h: ICC = 0.822  IMUs vs. OMC  12km/h: ICC = 0.715  15km/h: ICC = 0.470  18km/h: ICC = 0.629  21km/h: ICC = 0.741 | - | - | - |  |
|  | García-Pinillos et al.,2019 [42] | Stryd™:  r = 0.820, ICC = 0.813 (0.292–0.927), r^2^ = 0.166  RunScribe™:  r = 0.831, ICC = 0.896 (0.799–0.944), r^2^ = 0.001 | Stryd™: 16 ms  RunScribe™: 16 ms | Stryd™: -15 ms  RunScribe™: -6 ms | - |  |
|  | Deflandre et al.,2018 [37] | OMC:  8 km/h: ICC = -0.75 (-0.89–-0.48)  16 km/h: ICC = -0.70 (-0.86–-0.40)  Optogait:  8 km/h: ICC = -0.82 (-0.92–-0.62)  16 km/h: ICC = -0.77 (-0.90–-0.51) | - | - | - |  |
|  | Zrenner et al.,2020 [55] | - | - | Cavity: -11.0 ms  Heel: -1.3 ms  Instep: -22.6 ms  Lateral: -1.7 ms | - |  |
| Flight time | Falbriard et al.,2018 [40] | - | - | 15–30 ms | - | Conflicting |
|  | Gindre et al.,2016 [43] | IMUs vs. Optojump  12 km/h: ICC = 0.672  15 km/h: ICC = 0.717  18 km/h: ICC = 0.781  21 km/h: ICC = 0.817  IMUs vs. OMC  12 km/h: ICC = 0.689  15 km/h: ICC = 0.655  18 km/h: ICC = 0.656  21 km/h: ICC = 0.771 | - | - | - |  |
|  | García-Pinillos et al.,2019 [42] | Stryd™:  r = 0.809, ICC = 0.807 (0.179–0.929), r^2^ = 0.033  RunScribe™:  r = 0.754, ICC = 0.857 (0.747–0.920), r^2^ = 0.003 | Stryd™: 16.5 ms  RunScribe™: 17 ms | Stryd™: 14.5 ms  RunScribe™: 3 ms | - |  |
|  | Deflandre et al.,2018 [37] | OMC:  8 km/h:ICC = -0.85 (-0.94–-0.68)  16 km/h: ICC = -0.79 (-0.91–-0.55)  Optogait:  8 km/h: ICC = -0.91 (-0.96–-0.78)  16 km/h: ICC = 0.75 (-0.89–-0.48) | - | - | - |  |
| Swing time | Falbriard et al.,2018 [40] | - | - | 15–30 ms | - | Very limited |
| Step time | Falbriard et al.,2018 [40] | - | - | 0 ms | - | Very limited |
| Stride time | Bergamini et al.,2012 [33] | Correlation: 0.07 |  |  |  | Limited |
|  | Koldenhoven and Hertel,2018 [45] | Left limb  ICC = 0.94  Right limb  ICC = 0.91 | Left limb: 6.7 ms  Right limb: 8.1 ms | Left limb: 8.1 ms  Right limb: 3.2 ms | Left limb: -11.6–27.7 ms  Right limb: -20.3–26.8 ms |  |
|  | Zrenner et al.,2020 [55] | - | - | Cavity: -0.5 ms  Heel: 0.0 ms  Instep: 0.4 ms  Lateral: 0.3 ms | - |  |
| Step length | García-Pinillos et al.,2019 [42] | Stryd™:  r = 0.938, ICC = 0.975 (0.955–0.986), r^2^ = 0.062  RunScribe™:  r = 0.957, ICC = 0.968 (0.943–0.982), r^2^ = 0.008 | Stryd™: 3.924 cm  RunScribe™: 4.338 cm | Stryd™: 1.157 cm  RunScribe™: -0.557 cm | - | Very limited |
| Stride length | Koldenhoven and Hertel,2018 [45] | Left limb: ICC = 0.86  Right limb: ICC = 0.8 | Left limb: 10 cm  Right limb: 10 cm | Left limb: 70 cm  Right limb: 80 cm | Left limb: -140–10 cm  Right limb: -120–-50 cm | Moderate |
|  | Deflandre et al.,2018 [37] | OMC:  8 km/h: ICC = 0.92 (0.81–0.97)  16 km/h: ICC = 0.86 (0.69–0.94)  Optogait:  8 km/h: ICC = 0.97 (0.92–0.99)  16 km/h: ICC = 0.99 (0.99–1.0) | - | - | - |  |
|  | Brahms et al.,2018 [34] | ICC = 0.955 (0.927–0.970)  r = 0.961 | 8.3 cm | -3.2 cm | -18.3–11.8 cm |  |
|  | Zrenner et al.,2018 [54] | - | Algorithm 1: 45.2 cm  Algorithm 2: 19.9 cm  Algorithm 3: 7.6 cm  Algorithm 4: 15.3 cm | Algorithm 1: 17.7 ± 57.3 cm  Algorithm 2: -0.5 ± 25.6 cm  Algorithm 3: 2.00 ± 14.1 cm  Algorithm 4: 2.5 ± 20.1 cm | - |  |
|  | Zrenner et al.,2020 [55] | - | - | Cavity: 0.3 cm  Heel: -8.3 cm  Instep: -5.6 cm  Lateral: -3.3 cm | - |  |
| Step frequency | Gindre et al.,2016 [43] | IMUs vs. Optojump  12 km/h: ICC = 0.863  15 km/h: ICC = 0.941  18 km/h: ICC = 0.925  21 km/h: ICC = 0.865  IMUs vs. OMC  12 km/h: ICC = 0.889  15 km/h: ICC = 0.946  18 km/h: ICC = 0.844  21 km/h: ICC = 0.860 | - | - | - | Moderate |
|  | García-Pinillos et al.,2019 [42] | Stryd™:  r = 0.932, ICC = 0.965 (0.937–0.980), r^2^ = 0.025  RunScribe™:  r = 0.945, ICC = 0.964 (0.915–0.983), r^2^ = 0.012 | Stryd™: 2.780 spm  RunScribe™: 2.525 spm | Stryd™: -0.090 spm  RunScribe™: -1.316 spm | - |  |
|  | Deflandre et al.,2018 [37] | OMC:  8 km/h: ICC = 0.92 (0.81–0.96)  16 km/h: ICC = 0.81 (0.58–0.91)  Optogait:  8 km/h: ICC = 0.97 (0.93–0.99)  16 km/h: ICC = 0.96 (0.90–0.98) | - | - | - |  |
|  | De Fontenay et al.,2020 [38] | Moov Now^TM^: r = 0.976  MilestonePod: r = 0.997  RunScribe^TM^: r = 0.999  Zoi: r = 0.998  TgForce: r = 0.955 | - | Moov Now^TM^: 2.26 spm  MilestonePod: 1.59 spm  RunScribe^TM^: 1.12 spm  Zoi: 0.93 spm  TgForce: 4.5 spm | Moov Now^TM^: ± 1.98 spm  MilestonePod: ± 1.44 spm  RunScribe^TM^: ± 0.92 spm  Zoi: ± 1.28 spm  TgForce: ± 6.13 spm |  |
| Running speed | Koldenhoven and Hertel,2018 [45] | Left limb  ICC = 0.43  Right limb  ICC = 0.73 | Left limb: 0.2 m/s  Right limb: 0.1 m/s | Left limb: 0.1 m/s  Right limb: 0.4 m/s | Left limb: -0.2–0.3 m/s  Right limb: -0.1–0.8 m/s | Moderate |
|  | Zrenner et al.,2018 [54] | - | Algorithm 1: 0.622 m/s  Algorithm 2: 0.272 m/s  Algorithm 3: 0.133 m/s  Algorithm 4: 0.216 m/s | Algorithm 1: 0.209 ± 0.782 m/s  Algorithm 2: 0.005 ± 0.350 m/s  Algorithm 3: 0.028 ± 0.252 m/s  Algorithm 4: 0.055 ± 0.285 m/s | - |  |
|  | Fox et al.,2019 [41] | Medium  Back:r = 0.634 (0.19, 0.90)  Chest:r = 0.489 (0.43, 1.08)  High  Back:r = 0.785 (−0.11, 0.53)  Chest:r = 0.674 (0.18, 0.82) | - |  | - |  |
|  | Konharn et al.,2016 [46] | Moderate: ICC = 0.88  Vigorous: ICC = 0.97 | Moderate: 0.197 m/s  Vigorous: 0.269 m/s |  |  |  |
|  | Zrenner et al.,2020 [55] | - | - | Cavity: 0.0 m/s  Heel: -0.1 m/s  Instep: -0.1 m/s  Lateral: 0.0 m/s | - |  |
| Knee angle in the sagittal plane | Cooper et al.,2009 [35] | - | 3.4° ± 1.1° | - | - | Moderate |
|  | Dorschky et al.,2019 [39] | r = 0.99 | 5.3° ± 3.0° | - | - |  |
|  | Li et al.,2020 [48] | Cross-correlation: 0.956 ± 0.02 | - | 2.60° ± 2.87° | - |  |
|  | Validation of an IMU Suit for Military-Based Tasks  Mavor et al.,2020 [12] | - | V_OPT_ vs. X_IMU_  Right: 7.52° ± 3.20°  Left: 7.15° ± 3.03°  V_OPT_ vs. V_IMU_  Right: 9.83° ± 4.51°  Left: 9.17° ± 3.63°  V_OPT_ vs. V_IMU-CAL_  Right: 9.91° ± 4.62°  Left: 9.30° ± 3.65° | - | - |  |
|  | Nüesch et al.,2017 [50] | CMC = 0.881 | Without offset correction  17.9° ± 4.4°  With offset correction  7.8° ± 3.5° | - | - |  |
| Knee angle at initial contact | Nüesch et al.,2017 [50] | - | 19.3° | - | - | Very limited |
| Max Knee flexion/extension angle during Stance | Wouda et al.,2018 [53] | r^2^ = 0.72 | - | 0.38° | -4.1°–4.9° | Very limited |
| Minimal knee angle | Nüesch et al.,2017 [50] | - | 13.2° | - | - | Very limited |
| First maximal knee angle | Nüesch et al.,2017 [50] | - | 20.0° | - | - | Very limited |
| Second maximal knee angle | Nüesch et al.,2017 [50] | - | 19.8° | - | - | Very limited |
| Knee ROM (first half stride) | Nüesch et al.,2017 [50] | - | 5.7° | - | - | Very limited |
| Knee ROM (second half stride) | Nüesch et al.,2017 [50] | - | 7.6° | - | - | Very limited |
| Knee joint in the frontal plane | Mavor et al.,2020 [12] | - | V_OPT_ vs. X_IMU_  Right: 4.73° ± 1.28°  Left: 4.97° ± 2.08°  V_OPT_ vs. V_IMU_  Right: 4.90° ± 1.29°  Left: 5.17° ± 2.13°  V_OPT_ vs. V_IMU-CAL_  Right: 4.89° ± 1.42°  Left: 4.57° ± 1.20° | - | - | Very limited |
| Knee joint in the horizontal plane | Mavor et al.,2020 [12] | - | V_OPT_ vs. X_IMU_  Right: 6.44° ± 1.95°  Left: 7.62° ± 3.12°  V_OPT_ vs. V_IMU_  Right: 6.52° ± 1.84°  Left: 7.77° ± 2.99°  V_OPT_ vs. V_IMU-CAL_  Right: 5.38° ± 1.23°  Left: 6.07° ± 1.82° | - | - | Very limited |
| Hip angle in the sagittal plane | Dorschky et al.,2019 [39] | r = 0.98 | 8.7° ± 3.2° | - | - | Moderate |
|  | Li et al.,2020 [48] | Cross-correlation: 0.683 ± 0.14 | - | 1.99° ± 2.22° | - |  |
|  | Mavor et al.,2020 [12] | - | V_OPT_ vs. X_IMU_  Right: 8.07° ± 4.24°  Left: 8.23° ± 3.82°  V_OPT_ vs. V_IMU_  Right: 8.43° ± 4.29°  Left: 8.57° ± 3.89°  V_OPT_ vs. V_IMU-CAL_  Right: 8.36° ± 4.11°  Left: 8.64° ± 4.00° | - | - |  |
|  | Nüesch et al.,2017 [50] | CMC = 0.538 | Without offset correction  27.6° ± 3.2°  With offset correction  5.3° ± 2.2° | - | - |  |
| Hip angle at initial contact | Nüesch et al.,2017 [50] | - | 36.1° | - | - | Very limited |
| Minimal hip angle | Nüesch et al.,2017 [50] | - | 25.7° | - | - | Very limited |
| First maximal hip angle | Nüesch et al.,2017 [50] | - | 33.2° | - | - | Very limited |
| Second maximal hip angle | Nüesch et al.,2017 [50] | - | 25.1 | - | - | Very limited |
| Hip ROM (first half stride) | Nüesch et al.,2017 [50] | - | 8.6° | - | - | Very limited |
| Hip ROM (second half stride) | Nüesch et al.,2017 [50] | - | 4.2° | - | - | Very limited |
| Hip joint in the frontal plane | Mavor et al.,2020 [12] | - | V_OPT_ vs. X_IMU_  Right: 3.95° ± 1.20°  Left: 4.07° ± 1.37°  V_OPT_ vs. V_IMU_  Right: 4.23° ± 1.14°  Left: 4.18° ± 1.35°  V_OPT_ vs. V_IMU-CAL_  Right: 3.72° ± 0.89°  Left: 4.09° ± 0.99° | - | - | Very limited |
| Hip joint in the horizontal plane | Mavor et al.,2020 [12] | - | V_OPT_ vs. X_IMU_  Right: 3.87° ± 1.04°  Left: 4.22° ± 1.44°  V_OPT_ vs. V_IMU_  Right: 4.03° ± 1.04°  Left: 4.35° ± 1.43°  V_OPT_ vs. V_IMU-CAL_  Right: 3.97° ± 1.17°  Left: 4.06° ± 1.43° | - | - | Very limited |
| Rearfoot ROM in the sagittal plane | Koska et al.,2018 [47] | - | - | 10 km/h: -3.1°  12 km/h: -3.8°  15 km/h: -5.9° | 10 km/h  Q_2.5_ = -7°; Q_97.5_ = 3.4°  12 km/h  Q_2.5_ = -7.6°; Q_97.5_ = 2.1°  15 km/h  Q_2.5_ = -11.1°; Q_97.5_ = 1.8° | Very limited |
| Rearfoot eversion ROM | Koska et al.,2018 [47] | - | - | 10 km/h: 2.3°  12 km/h: 2.6°  15 km/h: 2.5° | 10 km/h: ± 4.3°  12 km/h: ± 4.9°  15 km/h: ± 5.8° | Very limited |
| Rearfoot ROM in frontal plane | Zrenner et al.,2020 [55] | - | - | Cavity: 0.0°  Heel: 1.2°  Instep: 2.3°  Lateral: 1.4° | - | Very limited |
| Ankle angle in the sagittal plane | Kim et al.,2021 [44] | r = 0.821 | - | - | - | Moderate |
|  | Dorschky et al.,2019 [39] | r = 0.98 | 4.6° ± 1.7° | - | - |  |
|  | Li et al.,2020 [48] | Cross-correlation: 0.76 ± 0.16 | - | 2.08° ± 1.75° | - |  |
|  | Mavor et al.,2020 [12] | - | V_OPT_ vs. X_IMU_  Right: 6.59° ± 1.76°  Left: 7.34° ± 2.22°  V_OPT_ vs. V_IMU_  Right: 6.50° ± 1.99°  Left: 7.35° ± 2.37°  V_OPT_ vs. V_IMU-CAL_  Right: 6.50° ± 2.09°  Left: 7.23° ± 2.41° | - | - |  |
|  | Nüesch et al.,2017 [50] | CMC = 0.677 | Without offset correction  17.7° ± 5.4°  With offset correction  5.4° ± 3.6° | - | - |  |
| Ankle angle at initial contact | Nüesch et al.,2017 [50] | - | 14.4° | - | - | Very limited |
| Maximal ankle angle | Nüesch et al.,2017 [50] | - | 19.1° | - | - | Very limited |
| First minimal ankle angle | Nüesch et al.,2017 [50] | - | 17.5° | - | - | Very limited |
| Second minimal ankle angle | Nüesch et al.,2017 [50] | - | 18.5° | - | - | Very limited |
| Ankle dorsiflexion ROM | Nüesch et al.,2017 [50] | - | 5.3° | - | - | Very limited |
| Ankle plantarflexion ROM | Nüesch et al.,2017 [50] | - | 7.1° | - | - | Very limited |
| Ankle joint in the frontal plane | Kim et al.,2021 [44] | r = 0.835 | - | - | - | Moderate |
|  | Mavor et al.,2020 [12] | - | V_OPT_ vs. X_IMU_  Right: 6.67° ± 1.37°  Left: 6.11° ± 1.14°  V_OPT_ vs. V_IMU_  Right: 6.21° ± 1.42°  Left: 5.46° ± 0.83°  V_OPT_ vs. V_IMU-CAL_  Right: 5.68° ± 1.35°  Left: 5.34° ± 1.00° | - | - |  |
| Ankle joint axial in the horizontal plane | Mavor et al.,2020 [12] | - | V_OPT_ vs. X_IMU_  Right: 7.16° ± 2.58°  Left: 5.90° ± 1.84°  V_OPT_ vs. V_IMU_  Right: 6.21° ± 2.18°  Left: 5.93° ± 1.74°  V_OPT_ vs. V_IMU-CAL_  Right: 5.98° ± 2.13°  Left: 5.90° ± 1.69° | - | - | Very limited |

*CCC* concordance correlation coefficient; *CMC* coefficient of multiple correlation; *ICC* intraclass correlation coefficient; *LoA* limits of agreement; *OMC* optical measurement capture system; *RMSE* root mean square error; *r* pearson correlation coefficient; *r^2^* coefficient of determination; *SEM* standard error of measurement; *spm* steps per minute; *95%CI* 95% confidence interval; *ROM* range of motion. Shaded represents that outcome have been included in the meta-analysis.

Supplementary Table 2: Qualitative summary of reliability for biomechanical parameters.

| Outcome | Study | Results | | | | Evidence |
| --- | --- | --- | --- | --- | --- | --- |
|  |  | ICC (95%CI) | RMSE | CVs/CV% | SEM |  |
| Stance time | Ammann et al.,2016 [32] | 0.911～0.960 |  | 2.9%–3.8% |  | Moderate |
|  | Gindre et al.,2016 [43] | 12 km/h: 0.993  15 km/h: 0.877  18 km/h: 0.946  21 km/h: 0.973 | - | 12 km/h: 6.5%  15 km/h: 6.7%  18 km/h: 8.3%  21 km/h: 9.9% | - |  |
|  | Deflandre et al.,2018 [37] | 8 km/h: 0.92 (0.82–0.97)  12 km/h: 0.97 (0.94–0.99)  16 km/h: 0.86 (0.70–0.94) | - | 8 km/h: 8.3 ms  12 km/h: 8.9 ms  16 km/h: 11.9 ms | 8 km/h: 4.2 ms  12 km/h: 2.4 ms  16 km/h: 5.9 ms |  |
| Flight time | Gindre et al.,2016 [43] | 12 km/h: 0.936  15 km/h: 0.951  18 km/h: 0.983  21 km/h: 0.978 | - | 12 km/h: 4.6%  15 km/h: 4.8%  18 km/h: 5.2%  21 km/h: 5.2% | - | Very limited |
| Step length | Deflandre et al.,2018 [37] | 8 km/h: 0.93 (0.84–0.97)  12 km/h: 0.97 (0.94–0.99)  16 km/h: 0.86 (0.70–0.94) |  | 8 km/h: 8.3 cm  12 km/h: 8.9 cm  16 km/h: 11.9 cm | 8 km/h: 0.9 cm  12 km/h: 0.8 cm  16 km/h: 2.6 cm | Very limited |
| Stride length | Deflandre et al.,2018 [37] | 8 km/h: 0.76 (0.5–0.89)  12 km/h: 0.95 (0.87–0.98)  16 km/h: 0.89 (0.75–0.95) | - | 8 km/h: 4.2 cm  12 km/h: 4.4 cm  16 km/h: 5 cm | 8 km/h: 1.7 cm  12 km/h: 1.2 cm  16 km/h: 2.4 cm | Very limited |
| Step frequency | Gindre et al.,2016 [43] | 12 km/h: 0.944  15 km/h: 0.820  18 km/h: 0.835  21 km/h: 0.942 | - | 12 km/h: 4.4%  15 km/h: 3.9%  18 km/h: 4.1%  21 km/h: 4.1% | - | Moderate |
|  | Deflandre et al.,2018 [37] | 8 km/h: 0.79 (0.55–0.91)  12 km/h: 0.94 (0.86–0.98)  16 km/h: 0.87 (0.72–0.95) | - | 8 km/h: 4.1 spm  12 km/h: 4.4 spm  16 km/h: 5.1 spm | 8 km/h: 3.1 spm  12 km/h: 1.8 spm  16 km/h: 3.3 spm |  |
| Running speed | Konharn et al.,2016 [46] | 0.97 |  |  |  | Very limited |
| Knee angle in the sagittal plane | Nüesch et al.,2017 [50] | - | 5.3° ± 3.1° | - | - | Very limited |
| Knee angle at initial contact | Nüesch et al.,2017 [50] | - | 1.4° | - | - | Very limited |
| Minimal knee angle | Nüesch et al.,2017 [50] | - | 4.9° | - | - | Very limited |
| First maximal knee angle | Nüesch et al.,2017 [50] | - | 5.4° | - | - | Very limited |
| Second maximal knee angle | Nüesch et al.,2017 [50] | - | 8.8° | - | - | Very limited |
| Knee ROM (first half stride) | Nüesch et al.,2017 [50] | 0.636 | 3.9° | - | - | Very limited |
| Knee ROM (second half stride) | Nüesch et al.,2017 [50] | 0.747 | 9.1° | - | - | Very limited |
| Ankle angle in the sagittal plane | Kim et al.,2021 [44] | 0.974 | - | - | 4.89° | Moderate |
|  | Nüesch et al.,2017 [50] | - | 6.7° ± 4.1° | - |  |  |
| Ankle angle at initial contact | Nüesch et al.,2017 [50] | - | 6.1° | - | - | Very limited |
| Maximal ankle angle | Nüesch et al.,2017 [50] | - | 3.7° | - |  | Very limited |
| First minimal ankle angle | Nüesch et al.,2017 [50] | - | 2.1° | - | - | Very limited |
| Second minimal ankle angle | Nüesch et al.,2017 [50] | - | 10.1° | - | - | Very limited |
| Ankle dorsiflexion ROM | Nüesch et al.,2017 [50] | 0.815 | 2.8° | - | - | Very limited |
| Ankle plantarflexion ROM | Nüesch et al.,2017 [50] | 0.355 | 10.4° | - | - | Very limited |
| Ankle angle in the frontal plane | Kim et al.,2021 [44] | 0.9 | - | - | 4.89° | Very limited |
| Hip angle in the sagittal plane | Nüesch et al.,2017 [50] | - | 3.8° ± 2.4° | - | - | Very limited |
| Hip angle at initial contact | Nüesch et al.,2017 [50] | - | 3.5° | - | - | Very limited |
| Minimal hip angle | Nüesch et al.,2017 [50] | - | 5.3° | - | - | Very limited |
| First maximal hip angle | Nüesch et al.,2017 [50] | - | 2.7° | - | - | Very limited |
| Second maximal hip angle | Nüesch et al.,2017 [50] | - | 3.8° | - | - | Very limited |
| Hip ROM (first half stride) | Nüesch et al.,2017 [50] | 0.810 | 4.0° | - | - | Very limited |
| Hip ROM (second half stride) | Nüesch et al.,2017 [50] | 0.914 | 3.9° | - | - | Very limited |

*CV* coefficient of variation; *ICC* intraclass correlation coefficient; *RMSE* root mean square error; *SEM* standard error of measurement; *spm* steps per minute; *95%CI* 95% confidence interval; *ROM* range of motion. Shaded represents that outcome have been included in the meta-analysis.
